# Robust prioritization of genomic features with stability selection

**DOI:** 10.1093/bioinformatics/btag398

**Published:** 2026-06-17

**Authors:** Gongshun Yang, Xi Lu, Cen Wu

**Affiliations:** Department of Statistics, Kansas State University, Manhattan, KS, United States; Department of Pharmaceutical Health Outcomes and Policy, College of Pharmacy, University of Houston, Houston, TX, United States; Department of Statistics, Kansas State University, Manhattan, KS, United States

## Abstract

**Motivation:**

The heterogeneity of complex diseases including cancer leads to heavy-tailed distributions in the disease traits. In such settings, non-robust variable selection methods are inherently susceptible to data contamination and can yield unstable or misleading results. This vulnerability becomes more severe for recently proposed approaches that introduce pseudo-features as negative controls, as these methods further amplify the curse of dimensionality by expanding the genotype matrix in the presence of outliers and high-dimensional genomic features.

**Results:**

We develop a robust variable selection framework with stability selection to prioritize genomic features in the presence of contamination. In contrast to existing approaches that rely on pseudo-features for error control, the proposed method achieves double robustness. First, it adopts least absolute deviation (LAD) LASSO to ensure robustness against outliers and heavy-tailed errors in disease traits. Second, it avoids augmenting the genotype matrix with pseudo-features, thereby mitigating the curse of dimensionality that is particularly problematic in high-dimensional genomic data. The proposed method has been extensively evaluated in simulation studies to demonstrate its effectiveness over multiple competing methods for variable selection. In addition, we have applied the proposed method and competing approaches to two real-data case studies: the The Cancer Genome Atlas (TCGA) Skin Cutaneous Melanoma (SKCM) dataset and an eQTL dataset. The results demonstrate that the proposed method achieves superior performance by identifying genomic features with higher reproducibility.

**Availability and implementation:**

The source code for implementing the proposed methods is publicly available at https://github.com/cenwu/RSS with an archival DOI https://doi.org/10.6084/m9.figshare.32306883.

## 1 Introduction

In genomics studies, phenotypic traits often demonstrate heavy-tailed distributions and outliers, reflecting underlying disease heterogeneity. For example, in cancer research, such heterogeneity is partly driven by the coexistence of dominant cancer subtypes with smaller, less prevalent subtypes, leading to contamination in the complex disease traits. We consider two gene expression datasets analyzed in the case studies of this paper. The first is the The Cancer Genome Atlas (TCGA) Skin Cutaneous Melanoma (SKCM) dataset, with log-transformed Breslow depth serving as the disease trait. The second is an eQTL dataset obtained from laboratory rats to study gene regulation in the mammalian eye, with TRIM32 gene expression used as the disease trait. Figure S1, available as supplementary data at *Bioinformatics* online in the supplementary file displays the histograms of the two traits, demonstrating that, both traits deviate substantially from normality. Furthermore, in the presence of high-dimensional genomics features, robust variable selection is essential for accommodating heavy-tailed distributions and outliers when identifying important omics features associated with disease phenotypes (Wu and Ma 2015).

Penalized variable selection methods are formulated within the “unpenalized loss + penalty function” framework where the unpenalized loss measures prediction error and the penalty function imposes shrinkage on regression coefficients corresponding to omics features. When a regression coefficient is shrunk to zero, the associated omics feature is effectively excluded from association with the disease phenotype. Consequently, these methods perform regularized estimation and variable selection simultaneously. The extent of shrinkage depends on the choice of the tuning parameter, which controls the degree of regularization and, in turn, the sparsity of the selected omics features (Wu and Wang 2020). When the least squares loss is used, penalized variable selection methods are non-robust and widely applied in bioinformatics (Ma and Huang 2008). To improve robustness, alternatives such as the least absolute deviation (LAD) and quantile check loss functions have been proposed to handle heavy-tailed distributions and outliers when identifying key omics features associated with disease phenotypes (Wu and Ma 2015).

With complex genomic data, instability in variable selection has been widely acknowledged in the literature. For example, Meinshausen and Bühlmann (2010) have demonstrated that even a small perturbation in the choice of the tuning parameter, on the order of 0.01, can dramatically alter the graphical structure of gene expression networks pinpointed using the graphical LASSO. When widely used procedures such as cross validation or information criteria are adopted, the optimal tuning parameter may vary across runs, making it difficult to obtain the same or similar results. As a consequence, the identified omics features often suffer from poor reproducibility. Unfortunately, even with robust methods, such irreproducibility persists. Wang *et al*. (2012) have analyzed the aforementioned eQTL data from lab rats using quantile SCAD and reported that nearly 80% of the selected gene expression features had selection frequencies below 50% when the model was refitted on random partitions of the data.

In the literature, to address the challenge in tuning selection, Yang *et al*. (2020) have proposed a permutation-assisted LASSO tuning procedure (plasso) for GWAS that constructs permuted pseudo-SNPs as negative controls. The tuning parameter is chosen along the LASSO solution path so that no pseudo-SNPs enter the model before the true predictors, and this calibration is repeated across multiple permutations to identify phenotype-associated variants based on their selection frequencies beyond prespecified cutoff (e.g. 90%). The strategy of taking advantage of negative controls through pseudo features is rooted in the popular knock-off framework (Barber and Candès 2015, Candes *et al*. 2018). In particular, the final step guarantees that the selected features have high selection frequencies and are not sensitive to small variations in the LASSO tuning parameter. Despite its success, plasso suffers from two fundamental limitations. First, it is built upon LASSO, which is non-robust and sensitive to heterogeneous disease phenotypes with outliers. Second, by incorporating permuted pseudo-features, plasso doubles the dimensionality of the genotype matrix, aggravating the curse of dimensionality. This dimensional inflation is especially detrimental under outlier contamination and skewed phenotypic distributions.

These limitations motivate the development of a robust variable selection framework with stability selection for prioritizing important genomics features. We adopt the least absolute deviation (LAD) LASSO as the core robust penalized regression method, which is less sensitive to outliers and heavy-tailed phenotypic distributions (Wang *et al*. 2007). Importantly, rather than further increasing the dimensionality by introducing pseudo-features, we directly apply stability selection (Meinshausen and Bühlmann 2010) by repeatedly fitting the LAD-LASSO on subsamples of the original genotype matrix. Extensive numerical studies using both simulated data and real genomics datasets demonstrate the superior performance of the proposed method compared with multiple alternative approaches.

Stability selection is a data-splitting strategy that plays an important role in developing reliable inference procedures for non-robust variable selection methods (Fan *et al*. 2024). However, for robust variable selection approaches, to the best of our knowledge, their behavior under stability selection has not been systematically investigated in bioinformatics studies. Notably, although Wang *et al*. (2012) have established oracle properties for the quantile SCAD estimator, their case study indicates that the resulting findings are difficult to reproduce when the same model is refitted on subsamples of the original data. This observation reveals a potential tension between theoretical inference guarantees and practical variable selection procedures that primarily rely on prediction-based tuning. Our study represents an initial step toward understanding the interplay between statistical inference and variable selection in robust high-dimensional settings. In particular, by reanalyzing the same eQTL dataset considered in Wang *et al*. (2012), we demonstrate that the proposed methods yield substantially more reproducible findings.

## 2 Materials and methods

### 2.1 Data and model

In cancer genomics studies, consider disease phenotype, clinical covariates and genomics features $\left\{y_{i},C_{i},x_{i}\right\}$ measured from *n* subjects (i=1,…,n). Let $y={\left(y_{1},\ldots ,y_{n}\right)}^{⊤}$ denote the disease phenotype, such as body weight or Breslow’s depth. The (k+1)-dimensional vector $C_{i}={\left(C_{i0},C_{i1},\ldots ,C_{ik}\right)}^{⊤}$ represents clinical covariates, including variables such as sex and age, where the first element $C_{i0}=1$ corresponds to the intercept. Define the n×p matrix $x={\left(x_{1},\ldots ,x_{n}\right)}^{⊤}$ with the *p*-dimensional vector $x_{i}={\left(x_{i1},\ldots ,x_{ip}\right)}^{⊤}$ consisting of measurements of *p* genomics features for the *i*th subject. We adopt the following robust linear regression model to establish the association between disease phenotypes and a set of predictors comprising omics features and clinical covariates:


(1) $$y_{i}=\sum_{t=0}^{k}\alpha_{t}C_{it}+\sum_{j=1}^{p}\beta_{j}x_{ij}+\epsilon_{i},$$

Where $\alpha ={\left(\alpha_{0},\ldots ,\alpha_{k}\right)}^{⊤}$ is a k×1 vector of regression coefficients denoting intercept and clinical covariates, and the p×1 vector of regression coefficients $\beta ={\left(\beta_{1},\ldots ,\beta_{p}\right)}^{⊤}$ corresponds to the *p* genomics features. To accommodate robustness against disease heterogeneity that widely exists cancer genomics studies, we assume that model errors $\epsilon_{i}$’s follow heavy-tailed distributions.

To place model (1) within the context of high-dimensional genomic analyses aimed at identifying important associations between genomic features and cancer outcomes, we consider a shrinkage estimation framework, in the spirit of LASSO (Tibshirani 1996). Such a penalization approach enables simultaneous parameter estimation and variable selection. Specifically, if $\beta_{j}\neq 0$, the *j*th genomic feature is deemed to be associated with the disease phenotype y. Otherwise, if $\beta_{j}=0$, the *j*th feature is regarded as unrelated to the cancer outcome and is excluded from the final model.

With large scale cancer omics features, the robust linear regression model (1) naturally extends to the following least absolute deviation (LAD) LASSO (Wang *et al*. 2007):


(2) $$\underset{\beta }{\min}\sum_{i=1}^{n}|y_{i}-\sum_{t=0}^{k}\alpha_{t}C_{it}-\sum_{j=1}^{p}\beta_{j}x_{ij}|+\lambda \sum_{j=1}^{p}|\beta_{j}|,$$

Where λ>0 is a tuning parameter that controls the degree of shrinkage and, consequently, the sparsity of genomic features associated with disease traits. Larger values of λ yield sparser solutions to the penalized loss by selecting fewer features. The LAD loss in model (2) provides a robust measure of prediction error by downweighting the influence of outliers through the $\ell_{1}$ norm. In contrast, the least squares loss amplifies the contribution of outlying observations via squared residuals and is therefore non-robust and sensitive to departures from normality.

### 2.2 Relevant work and limitations

Selecting appropriate tuning parameters plays a central role in the performance of penalized variable selection methods (Fan and Lv 2010). In practice, the tuning parameter λ is commonly chosen using data driven approaches such as cross validation (CV) or information criterion based methods, including AIC and BIC, which aim to balance model fit and sparsity. However, in high-dimensional settings, these procedures can be unstable and do not naturally account for uncertainty in the tuning parameter, as small perturbations in the optimal λ may lead to substantial changes in the subset of selected genomic features.

To overcome these disadvantages, Yang *et al*. (2020) have developed LASSO with a permutation-assisted procedure, or plasso, for genetic variants selection and prioritization, which is rooted in the knock–off variable selection utilizing pseudo-variables (Barber and Candès 2015, Candes *et al*. 2018). Without loss of generality, we ignore the clinical covariates for now and briefly summarize the central idea of Yang *et al*. (2020) as follows. First, construct a noisy copy of the original genetic variant matrix x, denoted by $x^{c}$, by permuting the rows of x without utilizing the phenotypic information. Second, fit LASSO on the augmented design matrix $x^{A}=[x,\;x^{c}]$. Third, along the LASSO regularization path, identify the smallest λ at which any estimated shrinkage coefficient corresponding to a permuted (null) feature in $x^{c}$ becomes nonzero, and select the penalty level immediately preceding this point as a data-adaptive tuning choice. Record the set of selected features at this penalty level. Fourth, repeat step 1 to step 3 multiple times and compute the selection frequency of each genetic variant. Finally, retain features whose selection frequency exceeds a prespecified empirical threshold (e.g. 90%), thereby favoring stable and reproducible signals over chance selections.

Yang *et al*. (2020) have demonstrated the superior performance of the permutation-assisted LASSO (plasso) in identifying SNPs that drive genotype–phenotype associations. Nevertheless, plasso may be less suitable for genomics studies involving heavy-tailed disease traits and high-dimensional omics features, as it relies on the standard LASSO and is therefore sensitive to long-tailed phenotypic distributions. Moreover, the use of pseudo-variables as negative controls expands the original n×p genotype matrix to n×2p, increasing the complexity of penalized model fitting under heavy-tailed errors due to the curse of dimensionality. In addition, estimating selection frequencies requires repeatedly refitting penalized models on the augmented n×2p genotype matrix across multiple permutations, which poses additional challenges for stability and reliability in high-dimensional settings with outliers.

Therefore, we propose a stability selection-assisted robust LASSO (RLSS) to improve robustness compared with plasso. The RLSS adopts the LAD-LASSO for model fitting and is thus less sensitive to outliers. However, despite its robustness, LAD-LASSO is well known to be instable and to produce a large number of false positives. This behavior arises in part because small changes in the tuning parameter can lead to dramatic variations in the selected set of cancer genomics features, which is typically observed in robust variable selection with non-differentiable penalized loss functions inducing additional optimization challenges in model estimation, in contrast to the simple soft-thresholding form of the solution obtained under standard LASSO. Accordingly, we further improve the reliability of LAD-LASSO by incorporating stability selection (Meinshausen and Bühlmann 2010, Shah and Samworth 2013) to robustly prioritize cancer genomics features, while avoiding the introduction of pseudo-variables that would otherwise expand the genotype matrix to one with dimensionality n×2p such as in Yang *et al*. (2020).

### 2.3 Robust prioritizing genomics features with stability selection

**Figure 1:**
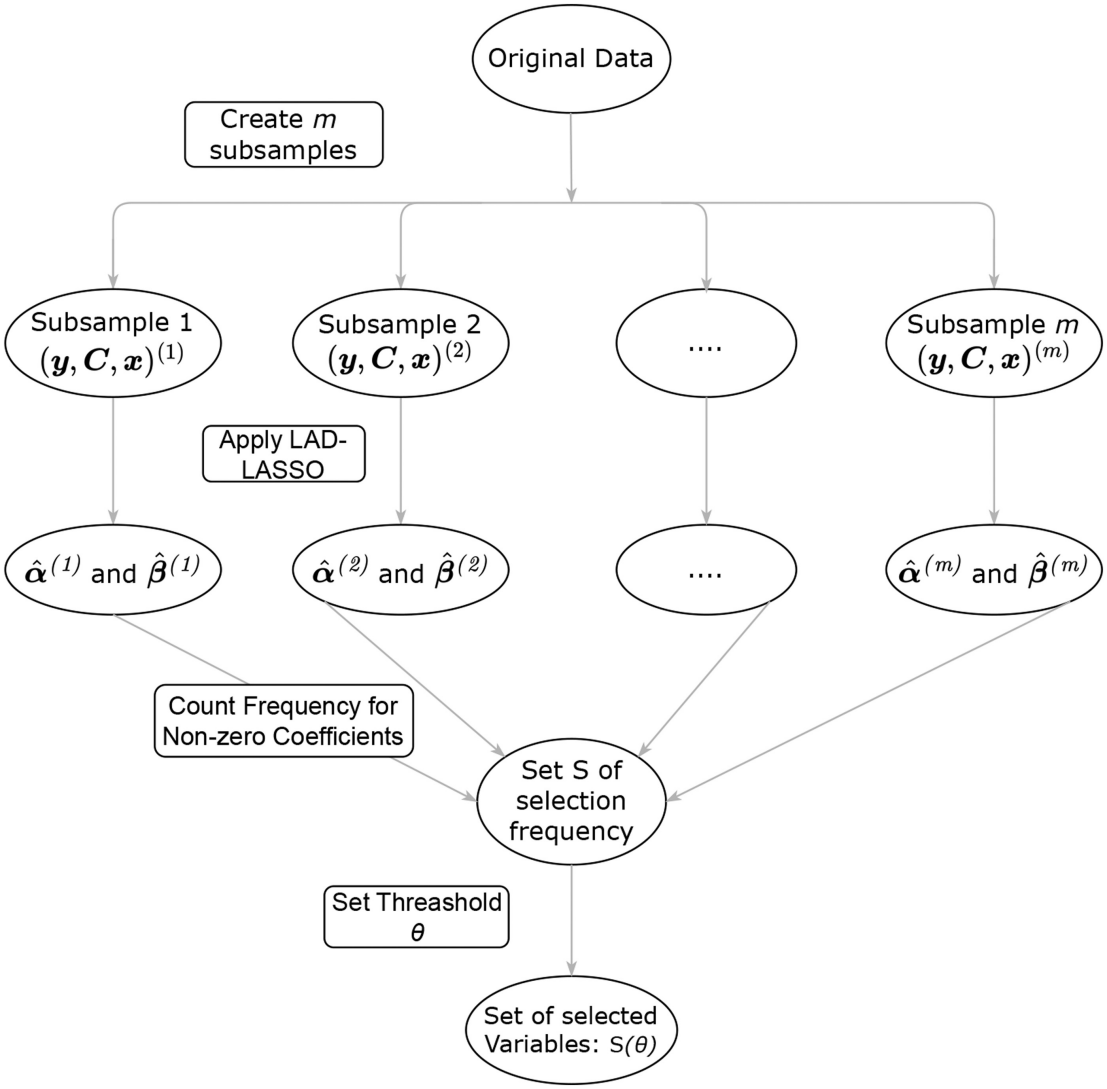
Procedures of robust feature selection with stability selection.

With a slight abuse of notation, we use ${\left\{y_{i},C_{i},x_{i}\right\}}_{i=1}^{n}$ and (y,C,x) interchangeably to denote measurements collected from *n* subjects, where $y\in R^{n\times 1}$ represents the disease trait, $C\in R^{n\times (k+1)}$ denotes the clinical covariates, and $X\in R^{n\times p}$ corresponds to the cancer genomics features. Consequently, the original dataset has overall dimensionality n×(k+2+p). Figure 1 shows the flowchart of the proposed robust feature selection with stability selection, which are described in detail below.

First, we randomly sample *nq* rows from the original dataset (y,C,x) without replacement, where q∈(0,1), resulting in a subsample of dimension nq×(k+2+p). This subsampling step is repeated *m* times, yielding *m* subsample datasets. We denote the *l*th subsample by ${\left(y,C,x\right)}^{\left(l\right)}$ for l=1,…,m.

Second, we apply LAD-LASSO to each of the *m* subsample dataset. For each subsample ${\left(y,C,x\right)}^{\left(l\right)}$, LAD-LASSO yields a sparse coefficient vector ${\hat{\beta }}^{\left(l\right)}$ corresponding to cancer genomics features. Within each subsample, a feature is assigned a value of 1 if it is selected (i.e. has a nonzero coefficient) and 0 otherwise. These binary indicators are then aggregated across all subsamples to quantify the selection frequency of each predictor. Specifically, the selection frequency of the *j*-th feature across the *m* subsamples is given by $f_{j}=\sum_{l=1}^{m}I({\hat{\beta }}_{j}^{\left(l\right)}\neq 0)$. Clinical covariates and the intercept are not subject to selection. Consequently, all components of ${\hat{\alpha }}^{\left(l\right)}$ are non-zero and included in every subsample.

We define the collection of selection frequencies for all cancer genomics features as


$$S=\left\{f_{j}=\sum_{l=1}^{m}I\left({\hat{\beta }}_{j}^{\left(l\right)}\neq 0\right):j=1,2,\ldots ,p\right\}.$$

Each $f_{j}\in S$ takes a value between 0 and *m* and represents how frequently the corresponding predictor is selected across the *m* subsamples. Predictors with larger values of $f_{j}$ are regarded as more important.

Finally, we select a threshold θ∈(0,1) to determine the importance of predictors based on their stability selection frequencies. A predictor is retained in the final selected set W(θ) if its selection ratio across the *m* subsamples exceeds the threshold θ. For example, the selection ratio of the *j*th predictor is given by $\frac{1}{m}\sum_{l=1}^{m}I\left({\hat{\beta }}_{j}^{\left(l\right)}\neq 0\right).$ Cancer genomics features with average selection frequencies greater than or equal to θ are retained, while the remaining predictors are excluded. This procedure favors predictors that are consistently selected across subsamples and thus improves the reliability of feature prioritization.

The formal selection rule is given by


(3) $$W(\theta )=\left\{x_{j}:j=1,2,\ldots ,p\;\text{such}\;\text{that}\;\frac{1}{m}\sum_{l=1}^{m}I\left({\hat{\beta }}_{j}^{\left(l\right)}\neq 0\right)\geq \theta \right\}.$$

Here, W(θ) denotes the set of selected predictors for a given threshold θ; *p* is the total number of predictors; *m* is the number of subsamples; I(·) is the indicator function; and ${\hat{\beta }}_{j}^{\left(l\right)}$ is the coefficient associated with the *j*th predictor estimated from the *l*th subsample.

In general, the proposed RLSS proceeds by repeatedly subsampling the data, applying LAD-LASSO to each subsample, and then ranking or selecting genomics features according to their empirical selection frequencies across all subsamples. The underlying premise of RLSS is that truly informative features are supposed to be selected consistently across subsamples, whereas spurious features are selected only sporadically. Consequently, the reliability of feature prioritization is assessed by the frequency with which predictors are selected across subsamples. In Section 2 of the Supplementary Material, available as supplementary data at *Bioinformatics* online, we provide the detailed computational algorithm for the LAD–LASSO used in RLSS, along with a summary of computational time. In the simulation study, we will show the performance of RLSS is insensitive to the subsample fraction *q* and the selection threshold θ as long as they are reasonably chosen.

Although both LAD–LASSO and stability selection are individually well established in the literature, the motivation for combining them is driven by the substantial instability of LAD–LASSO tuning in high-dimensional genomic studies with heterogeneous disease traits. Table S4, available as supplementary data at *Bioinformatics* online in the Appendix reveals that, even a small change in the tuning parameter can lead to dramatically different sets of selected variables, indicating the sensitivity and limited reproducibility of LAD–LASSO. Despite the practical importance of this issue, the instability of robust sparse estimation has received relatively little attention in the literature, even though published studies have reported poor reproducibility of robust variable selection methods with oracle properties (Wang *et al*. 2012) and valid inference procedures (Fan *et al*. 2026). The proposed RLSS framework addresses this challenge by incorporating stability selection as an effective mechanism to stabilize variable ranking and improve reproducibility across subsamples. Rather than relying on a single tuning choice that may produce unstable selections due to random variation, RLSS aggregates selection frequencies across repeated subsampling procedures, leading to more stable and robust genomic feature prioritization under heavy-tailed model errors.

### 2.4 Simulation

We have conducted a comprehensive evaluation of the proposed Robust LASSO with stability selection (RLSS) and compared it with five alternative methods: Robust LASSO (also known as LAD–LASSO; Wang *et al*. 2007), Robust LASSO with permutation-assisted tuning (RLP), LASSO with stability selection (Meinshausen and Bühlmann 2010), standard LASSO, and LASSO with permutation-assisted tuning (LP; Yang *et al*. 2020). Among these, RLP, the robust extension of LP (Yang *et al*. 2020), is also newly proposed. A summary of all methods is provided in Section 3 in the Appendix, available as supplementary data at *Bioinformatics* online. For both RLSS and LSS, we set the subsample fraction *q* to 0.7, the selection threshold to θ=90%, and the number of subsamples to m=80. A feature is therefore selected if it appears in more than 90% of the subsamples, corresponding to at least 72 selections. We note that the 90% cutoff to select features has also been used in Yang *et al*. (2020).

We generate cancer genomics features in 2 ways: (1) from a multivariate normal distribution with an AR(1) correlation of ρ=0.5, and (2) by directly sampling from the Skin Cutaneous Melanoma (SKCM) data analyzed in the case study. Responses are generated under both a homogeneous error model, $y_{i}=1+\sum_{j=1}^{k}\beta_{j}x_{ij}+\epsilon_{i}$, and a heterogeneous error model, $y_{i}=1+\sum_{j=1}^{k}\beta_{j}x_{ij}+(1+x_{ij})\epsilon_{i}$ where *k*=20 denotes the number of important features. The nonzero coefficients $\beta_{j}$ are simulated as either 0.3 or −0.3 with equal probability. We also consider two data dimensions (n,p)=(500,1000) and (1000,2000). The error term $\epsilon_{i}$ follows one of five distributions: N(0, 1)(Error 1),10%N(0,4) + 90%N(0,1) (Error 2), LogNormal(0,1) (Error 3), 90%N(0,1) + 10%Cauchy(0, 1) (Error 4), t(2) (Error 5). Except Error 1, Error 2 to Error 5 are heavy-tailed distributions. Their performance of all methods under comparison is assessed based on variable selection accuracy in true positive (TP), true negatives (TN), F1 score and Matthews Correlation Coefficient (MCC).

Table 1 shows the variable selection results of all the 6 methods under the homogeneous model with a dimension of n=1000,p=2000. Under heavy-tailed errors, it can be observed that RLSS consistently outperforms the rest, especially LP (Yang *et al*. 2020) and its robust extension RLP. For example, under the t(2) error (Error 5), the proposed RLSS achieves the highest performance, attaining both an F1 score and an MCC of 0.96 (*sd* 0.03). In contrast, RLP performs substantially worse, with an F1 score of 0.57 (*sd* 0.16) and an MCC of 0.63 (*sd* 0.13). The LP method performs the poorest, identifying on average only 3.07 (*sd* 2.85) of the 20 true positives. In addition, both RL and LASSO yield inferior identification results, indicating inferior performance when the tuning selection procedure is not adequately controlled. Please refer to a more detailed justification in Section 4.1 of the Appendix, available as supplementary data at *Bioinformatics* online.

**Table 1:** Evaluation of the homogeneous model under AR(1) correlation (n,p)=(1000,2000) with 100 replicates.

		RLSS	RL	RLP	LSS	LASSO	LP
*Error 1*	TP	20.00 (0.00)	20.00 (0.00)	16.73 (2.41)	20.00 (0.00)	20.00 (0.00)	19.93 (0.25)
	TN	1977.50(1.94)	1711.03(2.02)	1980.00(0.00)	1978.90(0.96)	1902.63(24.53)	1979.93(0.25)
	F1	0.94 (0.04)	0.15 (0.08)	0.91 (0.08)	0.97 (0.02)	0.35 (0.07)	1.00 (0.01)
	MCC	0.94 (0.05)	0.26 (0.07)	0.91 (0.07)	0.97 (0.02)	0.45 (0.06)	1.00 (0.01)
*Error 2*	TP	19.93 (0.37)	19.93 (0.18)	12.97 (3.11)	15.87 (2.65)	20.00 (0.00)	16.80 (2.20)
	TN	1979.07(1.01)	1850.20(2.23)	1979.90(0.31)	1979.97(0.18)	1900.70(24.18)	1979.97(0.18)
	F1	0.98 (0.02)	0.27 (0.10)	0.77 (0.13)	0.88 (0.09)	0.35 (0.07)	0.91 (0.07)
	MCC	0.98 (0.02)	0.38 (0.09)	0.79 (0.11)	0.88 (0.08)	0.45 (0.06)	0.91 (0.06)
*Error 3*	TP	20.00 (0.00)	19.20 (0.20)	14.03 (3.68)	14.40 (2.65)	19.27 (1.17)	8.33 (3.23)
	TN	1979.37(0.89)	1907.43(2.17)	1979.90(0.31)	1977.30(1.73)	1905.83(22.65)	1979.87(0.43)
	F1	0.98 (0.02)	0.38 (0.09)	0.81 (0.14)	0.77 (0.11)	0.35 (0.07)	0.57 (0.17)
	MCC	0.99 (0.02)	0.47 (0.08)	0.83 (0.12)	0.77 (0.10)	0.45 (0.06)	0.63 (0.13)
*Error 4*	TP	19.93 (0.25)	19.83 (0.15)	14.50 (2.61)	12.67 (6.05)	10.27 (9.36)	6.93 (7.87)
	TN	1978.93(1.14)	1896.67(3.12)	1979.90(0.31)	1975.43(6.60)	1941.43(39.50)	1979.90(0.31)
	F1	0.97 (0.03)	0.34 (0.07)	0.83 (0.09)	0.66 (0.26)	0.21 (0.17)	0.39 (0.42)
	MCC	0.97 (0.03)	0.44 (0.06)	0.84 (0.08)	0.68 (0.24)	0.26 (0.21)	0.42 (0.42)
*Error 5*	TP	18.90 (0.84)	19.97 (0.18)	8.37 (3.11)	7.37 (3.29)	13.17 (5.78)	3.07 (2.85)
	TN	1979.57(0.63)	1902.83 (2.32)	1979.93(0.25)	1975.53(3.12)	1940.47(25.13)	1979.83(0.59)
	F1	0.96 (0.03)	0.36 (0.07)	0.57 (0.16)	0.46 (0.18)	0.35 (0.14)	0.24 (0.21)
	MCC	0.96 (0.03)	0.46 (0.06)	0.63 (0.13)	0.47 (0.18)	0.39 (0.15)	0.31 (0.23)

**Figure 2:**
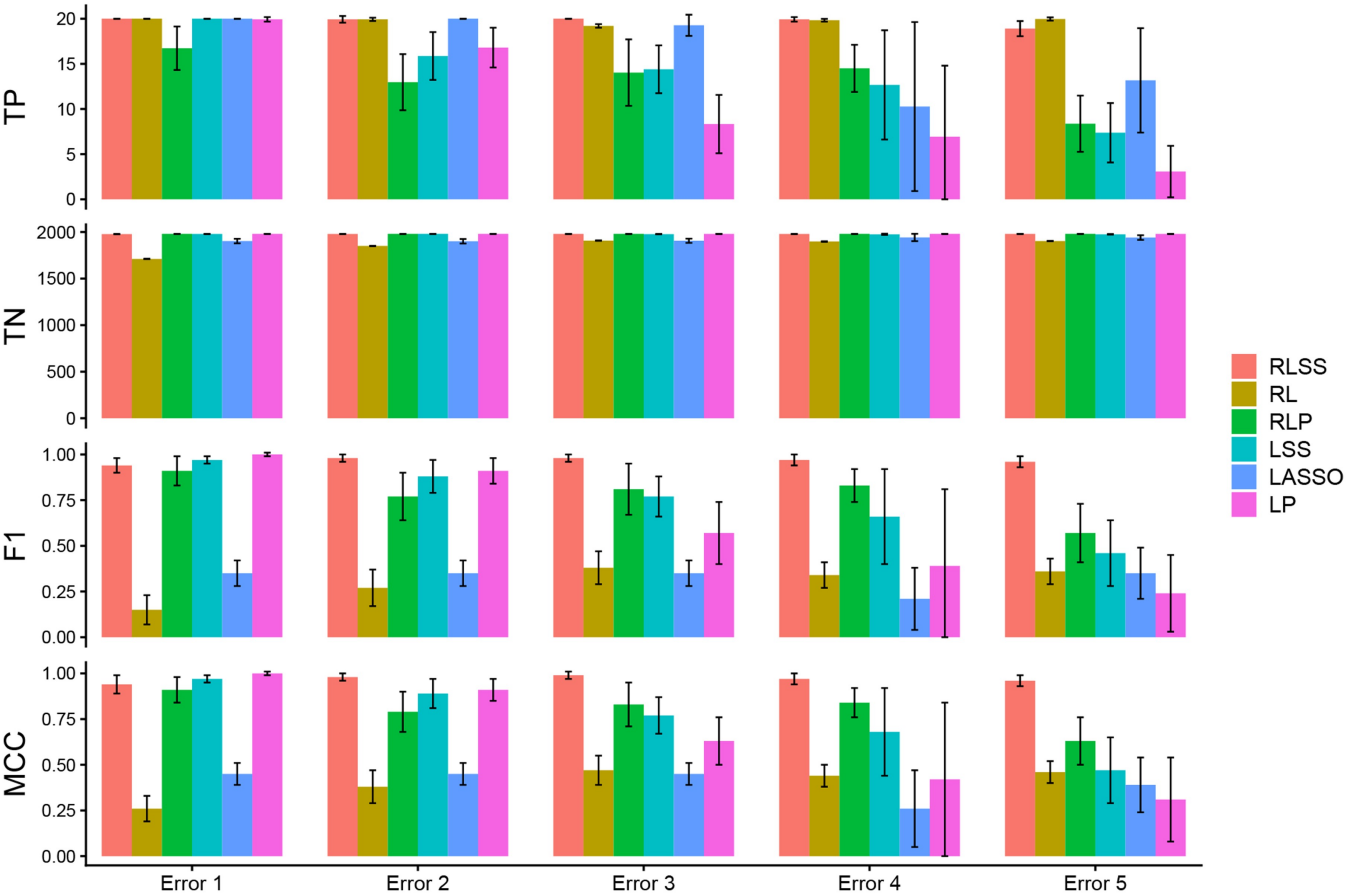
Performance under the homogeneous model error, AR(1) correlation and (*n,p*) = (1000, 2000) with 100 replicates. The corresponding results are given in Table 1.

The performance patterns of all six methods across the five error distributions in Table 1 are further visualized in Fig. 2. Under normal errors (Error 1), the F1 score and MCC of RLSS, RLP, and LP are comparable. However, as the error distribution becomes increasingly heavy-tailed, the superiority of RLSS is more evident. This superior performance is due to the *double robustness* of RLSS. First, robustness to heavy-tailed errors is achieved through the use of a robust penalized loss. While replacing LASSO with LAD-LASSO in LP (Yang *et al*. 2020) improves resistance to outliers, this robustification alone is insufficient. RLSS further achieves robustness to high dimensionality by avoiding augmentation of the genotype matrix. In contrast, RLP and LP introduce pseudo genomic features, thereby expanding the n×p genotype matrix to n×2p, which increases susceptibility to noise accumulation and instability in high-dimensional settings.

We have conducted additional simulation studies to further demonstrate the utility of RLSS. In the Appendix, available as supplementary data at *Bioinformatics* online, available as supplementary data at *Bioinformatics* online, we report variable selection results under the same data generating mechanism as in Table 1, but with reduced dimensionality (n,p)=(500,1000); these results are summarized in Table S5 and Figure S2, available as supplementary data at *Bioinformatics* online. Although the overall performance of all methods deteriorates in this setting with a limited sample size, RLSS continues to stand out and consistently outperforms the competing approaches. In addition, we generate genotype matrices by using the correlation matrix extracted from the SKCM gene expression data under the (n,p)=(500,1000) and (n,p)=(1000,2000) settings, with results reported in Tables S6 and S7, available as supplementary data at *Bioinformatics* online, respectively. Under more realistic correlation structures for generating the omics feature matrix, the performance of LP declines sharply, particularly in the presence of heavy-tailed errors, where both the F1 score and MCC of LP drop significantly to approximately 0.2–0.5, as shown in Tables S6 and S7 in the Appendix, available as supplementary data at *Bioinformatics* online. Similar conclusions regarding the superiority of RLSS can be made under the Table S8 with AR1 structure and heterogeneous model errors, and Table S9 and S10, available as supplementary data at *Bioinformatics* online under a higher p/n ratio with (n,p)=(1000,5000) and (500,2500), respectively.

Across this wide range of simulation settings, the superiority of RLSS over the alternative methods remains highly consistent. In particular, the superior performance of RLSS over RLP, a robust extension of LP (Yang *et al*. 2020), suggests the benefit of retaining the original genotype matrix rather than augmenting it with pseudo-features when model errors are heavy-tailed.

Interestingly, although LP is theoretically expected to outperform RLSS under normal errors (as shown in Table 1), we have observed a few settings in the Appendix, available as supplementary data at *Bioinformatics* online where RLSS performs slightly better even under Gaussian noise. This occurs mainly when (n,p)=(500,1000), whereas LP generally regains its advantage when (n,p)=(1000,2000). We conjecture that this is due to the curse of dimensionality caused by pseudo-feature augmentation. With a limited sample size of 500, the augmented dimension increases to 2000, which may weaken LP’s theoretical advantage. With a larger sample size (n=1000), LP better leverages its efficiency under Normal errors (denoted as Error 1) despite the augmented dimension (p=4000). These results further confirm the merit of RLSS in high-dimensional settings.

#### 2.4.1 Sensitivity analysis

In the proposed RLSS, the subsample fraction *q* and the selection stability threshold θ must be chosen carefully. We have conducted a sensitivity analysis over a range of combinations of q∈{70%,80%} and θ∈{50%,80%,90%}. The results are summarized in Tables S11–S17 in Section 4 from the Appendix, available as supplementary data at *Bioinformatics* online. We find that, for subsampling ratios q=70% and 80%, variable selection accuracy is comparable when the cutoff threshold θ is set to 80% or 90%, with θ=90% yielding slightly better performance. In contrast, a cutoff of θ=50% is overly permissive and results in a large number of false positives. Our findings are consistent with the conclusions from Meinshausen and Bühlmann (2010) which have shown that stability selection is generally insensitive to the choice of the subsampling fraction *q*, provided that *q* is sufficiently large for each subsample to be a representative approximation of the full dataset. These findings also support the 90% selection stability cutoff adopted in Yang *et al*. (2020).

## 3 Real data analysis

### 3.1 TCGA skin cutaneous melanoma data

In the case study, we first analyze TCGA SKCM data retrieved from the cBio Cancer Genomics Portal (Cerami *et al*. 2012). The disease trait of interest is the log-transformed Breslow’s depth. After matching genotypes with the phenotype, the resulting dataset consists of 294 subjects and 20 531 gene expression, along with age and gender as clinical covariates. Following Ren *et al*. (2023), we adopt a marginal linear model as a pre-screening procedure prior to downstream analysis. Marginal linear regression model based screening procedures are commonly used in ultra-high-dimensional studies for computational feasibility, including the widely used Sure Independence Screening framework of Fan and Lv (2008) among others. The top 600 genes with the smallest marginal *p*-values are retained for subsequent analysis.

**Figure 3:**
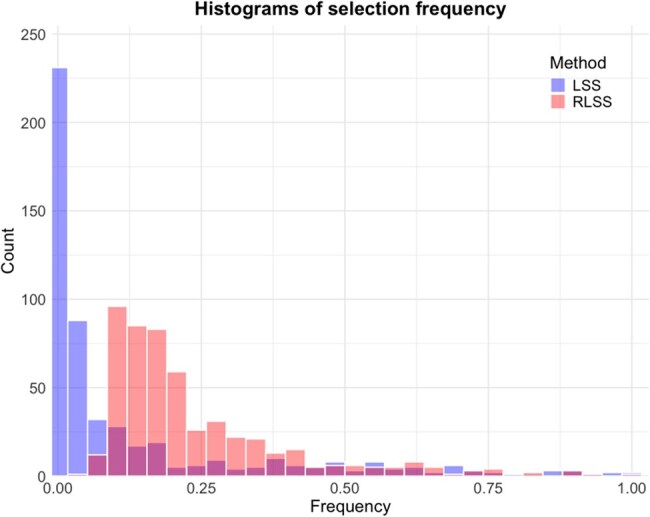
Histogram of selection frequencies for RLSS and LSS across all genes in the SKCM dataset.

The total number of identified gene expression features and their overlaps across all methods are summarized in the overlap table (Table S18, available as supplementary data at *Bioinformatics* online), while detailed estimation results are provided in Table S19 in the Appendix, available as supplementary data at *Bioinformatics* online. RLSS has identified 17 gene expressions. It can be observed that stability selection based procedures (RLSS and LSS) identify substantially different top-ranked genes compared with permutation-assisted procedures (RLP and LP). Moreover, Figure 3 presents histograms of selection frequencies for all genes under RLSS and LSS, illustrating that the robust method generally yields higher selection frequencies.

Our results provide empirical evidence supporting the biological relevance of the features selected by the proposed method. Notably, SEL1L3 has been identified as a key component of a tumor immune–relevant gene signature significantly associated with overall survival in melanoma patients, with demonstrated utility for prognosis, risk stratification, and prediction of response to ipilimumab immunotherapy (Mei *et al*. 2021). This finding suggests a potential role for SEL1L3 in immune regulation and melanoma progression. In addition, IER5, which is consistently up-regulated in melanoma, has been implicated in chemo-resistance and disease recurrence, indicating its involvement in treatment resilience and relapse, and emphasizing its promise as a therapeutic target (Wouters *et al*. 2012). Furthermore, FMNL2, also identified by RLSS, has been shown to drive melanoma progression and invasiveness, with elevated expression associated with poor clinical outcomes and functional evidence linking its suppression to reduced cell migration (Gardberg *et al*. 2016). Collectively, these findings underscore the ability of our approach to identify biologically meaningful genes with important prognostic and therapeutic implications in melanoma.

We further evaluate predictive performance using a multi-split testing procedure. Specifically, the 294 subjects are randomly partitioned into a training set (*n* = 200) and a testing set (*n* = 94). Each model is fitted on the training data and evaluated on the testing data across 100 random splits. Prediction accuracy is assessed using both the mean absolute error (MAE) and mean squared error (MSE). The proposed RLSS has demonstrated superior predictive performance compared to the rest, with MAE and MSE values of 0.68 (*sd* 0.08) and 0.79 (*sd* 0.29), respectively. In contrast, the alternative methods yielded higher prediction errors: RLP (MAE: 0.84, *sd* 0.07; MSE: 1.30, *sd* 0.35), LSS (MAE: 0.89, *sd* 0.07; MSE: 1.34, *sd* 0.39), LP (MAE: 0.84, *sd* 0.07; MSE: 1.30, *sd* 0.35), RL (MAE: 0.80, *sd* 0.07; MSE: 1.19, *sd* 0.29), and LASSO (MAE: 0.84, *sd* 0.08; MSE: 1.26, *sd* 0.32). The graphical representation of these results is provided in Figure S3 in Section 5 from the Appendix, available as supplementary data at *Bioinformatics* online.

### 3.2 GEO eQTL data

The dataset obtained from the Gene Expression Omnibus (GEO accession no. GSE5680) originates from an expression quantitative trait locus (eQTL) study in rats that investigates gene regulation in the mammalian eye and identifies genetic variants relevant to human ocular diseases (Scheetz *et al*. 2006). Whole-eye gene expression has been profiled using Affymetrix microarrays in 120 male F2 rats derived from an SR/JrHsd × SHRSP intercross, yielding expression measurements for 31 042 probes. Following standard pre-processing procedures to remove probes with low expression levels or low variability, 18 958 probes have been retained for subsequent analysis. The phenotype of interest is TRIM32, a gene reported to be associated with human hereditary diseases of the retina, corresponding to probe 1389163_at. The remaining gene expression measurements have been treated as potential predictors. Following the screening procedure of Wang *et al*. (2012), we have reduced the dimensionality of the data and retained 323 probes for downstream analysis, aiming to identify potential regulators of TRIM32 expression and to gain insight into the genetic architecture underlying eye-related disorders.

**Figure 4:**
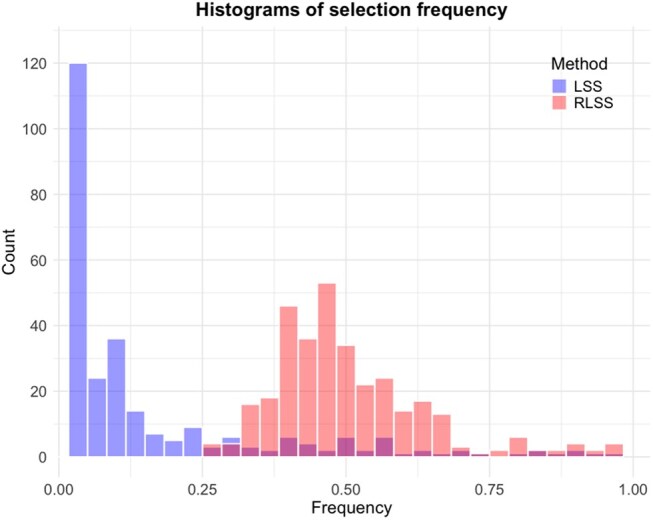
Histogram of selection frequencies for RLSS and LSS across all probes in the eQTL data.

RLSS identifies 14 probes. The numbers of selected probes and their overlaps across methods are summarized in Table S18 in the Appendix, available as supplementary data at *Bioinformatics* online, and the corresponding estimated coefficients are reported in Table S20, available as supplementary data at *Bioinformatics* online. The histograms of selection frequencies for all genes under RLSS and LSS (Fig. 4) indicate that RLSS yields higher selection frequencies, suggesting greater reproducibility of its findings. Without stability selection, Wang *et al*. (2012) and Fan *et al*. (2026) have shown that features identified by quantile SCAD with oracle properties and by robust Bayesian horseshoe regression with valid inference procedures have very low selection frequencies when the model is repeatedly fitted on random partitions of the eQTL data.

Prediction performance has been evaluated using the aforementioned multi-split strategy, with the 120 subjects randomly partitioned into training (*n* = 60) and testing (*n* = 60) sets across multiple replicates. Each method has been fitted on the training data and evaluated on the testing data. Boxplots of predictive accuracy are shown in Figure S4 in the Appendix, available as supplementary data at *Bioinformatics* online, which indicate that the RLSS leads to better prediction, with a predictive mean absolute error (MAE) of 0.057 (*sd* 0.012) and predictive mean squared error (MSE) of 0.0056 (*sd* 0.0013). This performance surpasses other methods including RL (MAE: 0.062, *sd* 0.0094; MSE: 0.0065, *sd* 0.0015), RLP (MAE: 0.065, *sd* 0.0067; MSE: 0.0085, *sd* 0.0019), LASSO (MAE: 0.065, *sd* 0.0089; MSE: 0.0073, *sd* 0.0014), LSS (MAE: 0.068, *sd* 0.0096; MSE: 0.0073, *sd* 0.0012), and LP (MAE: 0.073, *sd* 0.010; MSE: 0.0091, *sd* 0.0023).

## 4 Discussion

Besides RLSS, we have also developed RLP, which is the robust extensions of permutation-assisted LASSO (plasso; Yang *et al*. (2020)). RLP replaces LASSO by LAD-LASSO directly within the plasso framework. Empirically, RLP shows inferior performance compared with RLSS. This observation suggests that, in the presence of heterogeneous disease traits, the strategy of expanding the genotype matrix by introducing negative controls (i.e. null features) is less effective than under homogeneous and light-tailed error settings. The degradation in performance is likely attributable to the combined effects of high dimensionality and heavy-tailed or outlying responses, which jointly undermine the reliability of permutation-based negative controls.

The proposed robust variable selection with stability selection can be extended in the following aspects. First, LAD–LASSO cannot account for structured sparsity such as strong correlations among omics features, the network structure and the sparse group/bi-level structure. A natural extension of RLSS is to replace the LAD–LASSO fitting step with a robust structured penalized estimator while retaining the stability selection module. For example, robust sparse group LASSO can be adopted to encourage both group-level selection and within-group sparsity, allowing genomic features to be selected according to pathway, functional annotation, or other biologically meaningful group structures with stability measures. Such extensions will provide a pathway- or network-aware robust feature prioritization framework for high-dimensional omics studies. Second, the proposed framework can be generalized to other types of phenotypes beyond continuous cross-sectional outcomes, such as longitudinal and survival outcomes. We will postpone the investigations to the near future. Last but not least, we focus primarily on estimation in this study, which differs from knockoff-based variable selection frameworks that provide valid inferential guarantees but may suffer from limited stability. RLSS may be improved with statistical inference procedures. Please refer to Section 4.2 of the Appendix for a more detailed discussion between RLSS and knock-off frameworks.

## Supplementary Material

btag398_Supplementary_Data

## Data Availability

The Cancer Genome Atlas (TCGA) Skin Cutaneous Melanoma data are publicly available through the Genomic Data Commons (GDC) Data Portal at https://portal.gdc.cancer.gov/. The Gene Expression Omnibus (GEO) eQTL data can be accessed from the GEO database under accession number GSE5680.
