# Supplementary material for: Robust prioritization of genomic features with stability selection
Source: Bioinformatics. 2026 Jun 17;42(7):btag398. doi: 10.1093/bioinformatics/btag398 (PMC13353225; doi:10.1093/bioinformatics/btag398)
Supplement: btag398_Supplementary_Data [file btag398_supplementary_data.pdf]

Supplementary Material for "Robust prioritization of genomics features with stability selection"

Gongshun Yang[1a], **Xi Lu**[2a,*] **and Cen Wu**[1*]

[1] Department of Statistics, Kansas State University, Manhattan, KS, USA
[2] Department of Pharmaceutical Health Outcomes and Policy, College of Pharmacy, University of Houston, Houston, TX, USA
[a] Joint first authors
Corresponding author: wucen@ksu.edu, xlu20@uh.edu

1 Histogram

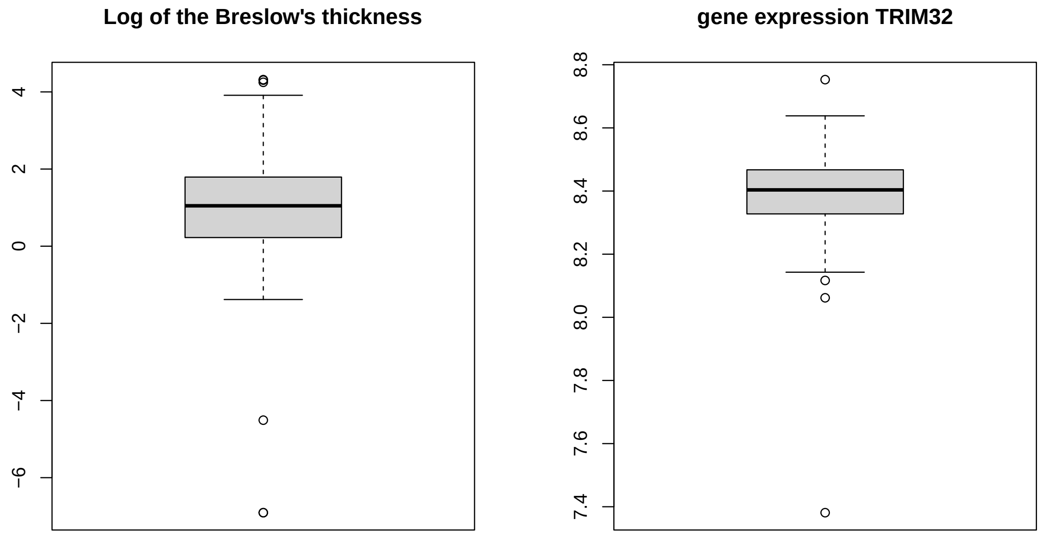

Figure S1: Distributions of the disease phenotype for the TCGA SKCM (left) and GEO eQTL (right) datasets.

2 Computation details for the proposed RLSS

2.1 Model fitting

Robust LASSO with stability selection, or RLSS, utilizes LAD-LASSO for model fitting, for which the computational algorithms have been thoroughly investigated in the literature[1–4], among others. In our study, we adopt the weighted median regression algorithm by fitting LAD-LASSO[2–4]. Specifically, the penalized loss function of LAD-LASSO, $g(\boldsymbol{\alpha}, \boldsymbol{\beta}) = \sum_{i=1}^{n} |y_i - \sum_{t=0}^{k} \alpha_t C_{it} - \sum_{j=1}^{p} \beta_j x_{ij}| + \lambda \sum_{j=1}^{p} |\beta_j|$, can be further expressed as:

$$
\begin{aligned}
g(\boldsymbol{\alpha}, \boldsymbol{\beta}) &= \sum_{i=1}^{n} |\frac{y_i - \sum_{t=0}^{k} \alpha_t C_{it}}{x_{ij}} x_{ij} - \sum_{j=1}^{p} \beta_j x_{ij}| + \lambda \sum_{j=1}^{p} |\beta_j| \\
&= \sum_{i=1}^{n} |x_{ij}||\frac{y_i - \sum_{t=0}^{k} \alpha_t C_{it} - \sum_{s \neq j}^{p} \beta_s x_{is}}{x_{ij}} - \beta_j| + \lambda \sum_{j=1}^{p} |\beta_j|,
\end{aligned} \tag{S1}
$$

where $\boldsymbol{\alpha}$ represent the coefficient vector of the intercept and clinical covariates. We update $\boldsymbol{\alpha}$ and $\boldsymbol{\beta}$ iteratively through weighted mediation regression. Given that $\boldsymbol{\alpha}$ has been updated, we can then estimate $\boldsymbol{\beta}$ in a component-wise manner through the following weighted median regression by fixing $\boldsymbol{\alpha}$:

$$
\beta_j^{(d)} = \text{argmin}_{\beta_j} \frac{1}{n+1} \sum_{i=1}^{n+1} w_{ij} u_{ij}, \tag{S2}
$$

where the weights w_{ij} and pseudo observations u_{ij} are defined as:

$$
u_{ij}^{(d)} = \begin{cases} \frac{y_i - \sum_{t=0}^{k} \alpha_t C_{it} - \sum_{s \neq j} \beta_s^{(d-1)} x_{is}}{x_{ij}} - \beta_j & i = 1, 2, \ldots, n \\ 0 - \beta_j & i = n+1, \end{cases} \tag{S3}
$$

and:

$$
w_{ij}^{(d)} = \begin{cases} |x_{ij}| & i = 1, 2, \ldots, n \\ \lambda & i = n+1. \end{cases} \tag{S4}
$$

The minimizer $\beta_j^{(d)}$ is the weighted median of $n+1$ pseudo observations u_{ij} with weights w_{ij}. The procedure will continue to iterate and update $\boldsymbol{\alpha}$ and $\boldsymbol{\beta}$ until convergence. We have implemtned the model fitting procedure in R package *regnet*[5]. The tuning parameters are chosen through 5-folds cross-validation.

Table S1: Computational Algorithms for RLSS

**Stability Selection Algorithm**
**Draw** a size of fraction q from original data without replacement randomly:<br>**Repeat** this process for m times independently to formulate m subsamples.<br>**For** each subsample of the data<br>Initialize $d = 0$, $\alpha^{(0)}$ and $\beta^{(0)}$;<br>**Repeat**<br>Update $\alpha^{(d+1)}$ component-wise using weighted median regression;<br>**for** $j = 1, 2, \ldots, p$<br>Compute u_j and w_j via equations (S3) and (S4);<br>Update $\beta_j^{(d+1)}$ using the weighted median in equation (S2);<br>Increment j by 1;<br>**end for**<br>Increment d by 1;<br>**until convergence**.
**End For**
Count the frequency of predictors that have non-zero coefficients across the m subsamples.<br>Choose a threshold θ.<br>Select final predictors via equation (3) in the main text.

Remark: Within the adapted stability selection framework, the parameters fraction q and threshold θ play important roles. The fraction q determines the subsample size and therefore directly affects the variability of the results. Meanwhile, the threshold θ specifies the criterion for predictor inclusion according to the consistency of selection across subsamples. Although the optimal choices of these parameters may vary across applications, careful specification of q and θ is important for ensuring the robustness and reliability of the resulting feature selection procedure.

2.2 Computational time

Table S2 summarizes the computational cost of all methods under comparison. As expected, RLSS incurs a heavier computational cost than LSS because RLSS relies on LAD-LASSO fitting implemented through the R package *regnet*[3], whereas LSS is based on standard LASSO implemented via the R package *glmnet*[6] which is widely recognized as one of the most efficient tools for fitting penalized regression models. Nevertheless, the computational burden remains affordable in practice. Under sequential implementation, the CPU time of RLSS ranges from approximately 298 seconds to 1909 seconds across all considered settings, while LSS ranges from 12 seconds to 47 seconds. The computational cost increases with both sample size and dimensionality. Although RLSS is slower than LSS, it remains computationally feasible for high-dimensional settings considered in this paper.

In addition, although stability selection requires repeated model fitting across all the subsamples, this procedure is naturally parallelizable since each subsample can be processed independently. As a result, parallel computation can substantially reduce the overall runtime. In Table S2, we report a rough estimate of the parallel runtime of RLSS and LSS by dividing the total computational time by the number of subsamples (80 in the simulation study). For example, under the setting ($n = 1000, p = 1500$), the runtime of RLSS decreases from 1909.13 seconds under sequential implementation to 23.86 seconds under parallel implementation. These results indicate that RLSS remains computationally feasible for high-dimensional genomics applications when implemented using modern parallel computing strategies.

Table S2: Computational time (CPU time in seconds) for all methods under comparison.

**Setting**	**Time**	**RLSS**	**RL**	**RLP**	**LSS**	**LASSO**	**LP**
$n = 500,\ p = 500$	Sequential	298.21	4.75	222.52	12.15	0.69	0.71
	Parallel	3.73	–	–	0.15	–	–
$n = 500,\ p = 1000$	Sequential	493.97	7.73	586.59	20.44	0.42	0.82
	Parallel	6.17	–	–	0.26	–	–
$n = 500,\ p = 1500$	Sequential	900.61	14.31	1408.94	31.87	0.53	1.38
	Parallel	11.26	–	–	0.39	–	–
$n = 1000,\ p = 500$	Sequential	851.57	11.39	516.54	44.39	0.58	5.82
	Parallel	10.64	–	–	0.55	–	–
$n = 1000,\ p = 1000$	Sequential	1352.84	20.79	1366.21	35.14	1.91	2.56
	Parallel	16.91	–	–	0.44	–	–
$n = 1000,\ p = 1500$	Sequential	1909.13	29.69	2633.16	47.08	1.22	3.57
	Parallel	23.86	–	–	0.59	–	–

3 Summary of all methods under comparison

Table S3: Summary of all methods under comparison.

		Methods	Reference
Robust	**RLSS**	Robust LASSO with stability selection	Newly proposed
	**RL**	Robust LASSO	Wang et al. (2007) [1]
	**RLP**	Robust LASSO with permutation	Newly proposed
Non-robust	**LSS**	LASSO with stability selection	Meinshausen et al. (2010) [7]
	**LASSO**	Original LASSO	Tibshirani. (1996) [8]
	**LP**	LASSO with permutation	Yang et al. (2020) [9]

4 Additional simulation results

4.1 Inappropriate tuning selections for RL and LASSO

To further demonstrate that the inferior and unstable variable selection performance of LASSO and RL is partially due to inappropriate tuning parameter selection, we have evaluated their variable selection performance under Error 2 ($10\%N(0,4)+90\%N(0,1)$). Table S4 shows that both methods produce substantially different variable selection results across the sequence of tuning parameters, particularly for RL. For example, when the tuning parameter changes by only 0.12, the number of true positives drops dramatically from 20 to 4.73, indicating that even a small perturbation in tuning parameter selection can severely affect the results. This observation further highlights the need for additional procedures to properly stabilize and control tuning parameter selection. For LASSO, the soft-thresholding rule is acknowledged to produce biased estimates and typically yields a relatively large number of false positives, leading to less reliable variable selection results.

Table S4: Evaluation of the performance of LASSO and RL with homogeneous model under $(n, p) = (500, 1000)$, AR(1) correlation with Error 2 across different tunings.

**LASSO**	λ_1=0.15	λ_2=0.17	λ_3=0.20	λ_4=0.24	λ_5=0.28	λ_6=0.30	λ_7=0.35
TP	18.93(1.05)	18.23(1.41)	16.93(2.26)	13.83(2.60)	11.03(2.25)	9.30(2.29)	5.63(1.87)
TN	943.70(7.92)	956.93(6.36)	968.83(4.09)	976.40(2.55)	978.83(1.05)	979.33(0.88)	979.87(0.35)
F1	0.51(0.07)	0.60(0.08)	0.71(0.09)	0.74(0.10)	0.68(0.09)	0.61(0.11)	0.43(0.11)
MCC	0.56(0.06)	0.63(0.07)	0.71(0.09)	0.74(0.10)	0.70(0.08)	0.65(0.09)	0.51(0.09)
**RL**	λ_1=0.05	λ_2=0.08	λ_3=0.10	λ_4=0.12	λ_5=0.14	λ_6=0.15	λ_7=0.17
TP	20.00(0.00)	19.90(0.30)	19.13(1.04)	16.10(2.41)	11.53(2.30)	9.03(1.67)	4.73(1.82)
TN	620.93(19.51)	885.33(10.96)	949.13(7.05)	971.27(2.81)	977.93(1.57)	979.10(1.03)	979.83(0.38)
F1	0.10(0.01)	0.30(0.03)	0.55(0.06)	0.72(0.08)	0.68(0.09)	0.60(0.08)	0.37(0.12)
MCC	0.18(0.01)	0.40(0.02)	0.60(0.05)	0.72(0.08)	0.69(0.09)	0.64(0.07)	0.47(0.10)

4.2 Comparisons to robust knock-off methods

The main appeal of the proposed RLSS lies in stability-based robust variable selection. It emphasizes a different perspective compared to knockoff methods which provide valid inferential guarantees on the False Discovery Rate (FDR). In the presence of heavy-tailed errors in disease phenotypes, even robust variable selection methods with valid inference guarantees may not necessarily yield reproducible results. For example, recent work on robust Bayesian regression with horseshoe-family priors has been shown to provide valid Bayesian credible intervals on finite samples (Fan et al. (2026)[10]). However, when applied to eQTL data with skewed disease traits, most findings are not reproducible. Similar observations regarding irreproducibility of findings have been reported for theoretically grounded regularized quantile regression applied to the same eQTL data in Wang et al. (2012)[11], as discussed in more detail in the Introduction.

We conjecture that, for variable selection approaches with valid inferential guarantees to yield reproducible findings, stability selection should be incorporated as a built-in component, as in Meinshausen and Bühlmann (2010)[7]. This is particularly important for robust variable selection methods, whose performance is highly sensitive to tuning choices, as illustrated in Table S4. To the best of our knowledge, we are not aware of any published robust knockoff methods with publicly available software for direct comparison with RLSS. A key limitation of knockoff procedures, particularly under heavy-tailed error distributions, is the doubling of the feature dimension through pseudo-feature augmentation, which can make them more vulnerable to the curse of dimensionality.

4.3 Simulations under AR(1) correlation and homogeneous errors

Table S5 and the corresponding Figure S2 present results for the AR(1) correlation setting with $(n, p) = (500, 1000)$. The overall performance patterns of all methods closely align with those reported in Table 1 in the main text. In this smaller scale setting, RLSS consistently outperforms RLP, LSS, and LP, while methods based on LAD-LASSO generally outperform their least-squares counterparts, particularly under heavy-tailed error distributions.

A comparison between Table S5 and Table 1 indicates an overall degradation in performance as both the sample size and dimensionality decrease. Despite this trend, methods that incorporate LAD-LASSO and stability selection retain relatively strong performance, suggesting greater robustness to reduced sample size and dimension. Among all competing approaches, the proposed RLSS shows the highest resilience to performance deterioration.

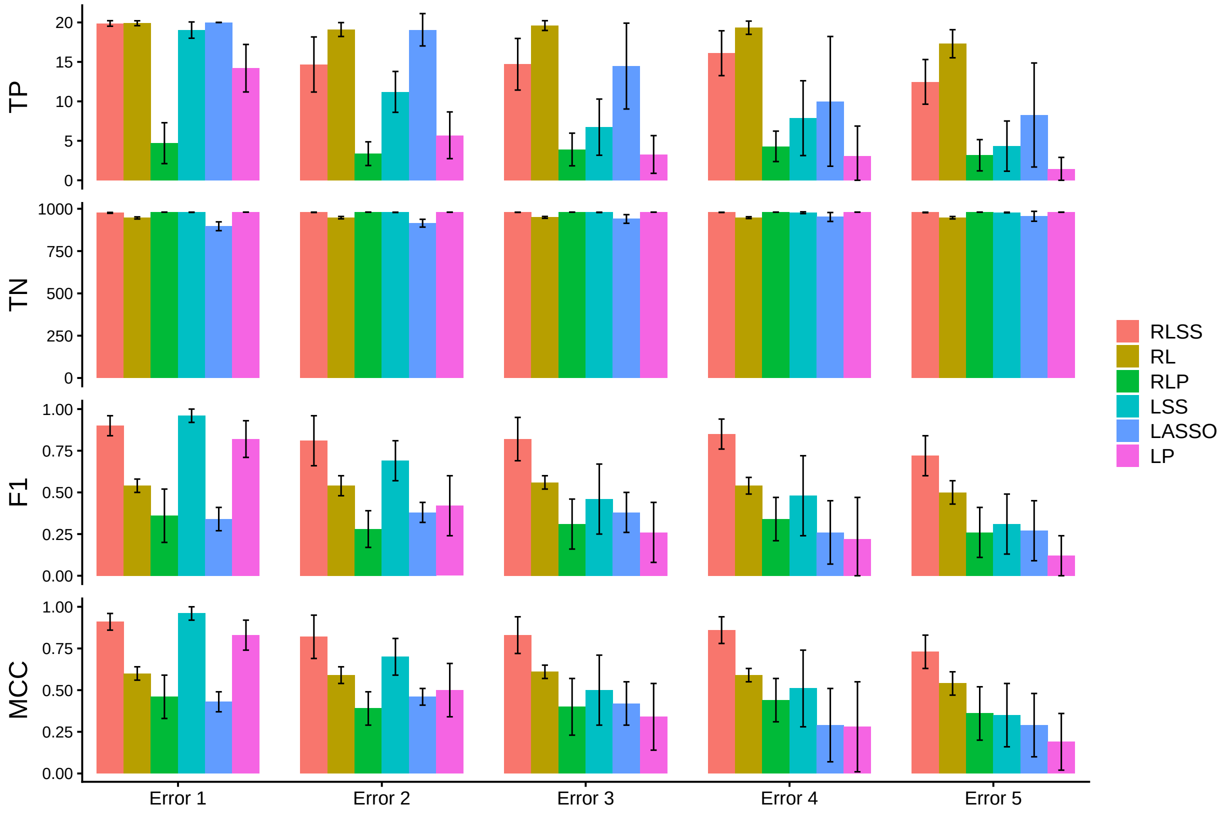

Figure S2: Performance under the homogeneous model error, AR(1) correlation and (n, p) = (500, 1000) with 100 replicates. The corresponding results are given in Table S5 in the Appendix.

Table S5: Evaluation of the homogeneous model under AR(1) correlation $(n, p) = (500{,}1000)$ with 100 replicates.

		RLSS	RL	RLP	LSS	LASSO	LP
**Error 1**	TP	19.87(0.35)	19.90(0.31)	4.70(2.59)	19.03(1.03)	20.00 (0.00)	14.20 (3.01)
	TN	975.80(2.72)	945.87(6.22)	979.93(0.25)	979.30(0.88)	896.57(26.09)	979.87(0.35)
	F1	0.90 (0.06)	0.54 (0.04)	0.36 (0.16)	0.96 (0.04)	0.34 (0.07)	0.82 (0.11)
	MCC	0.91 (0.05)	0.60 (0.04)	0.46 (0.13)	0.96 (0.04)	0.43 (0.06)	0.83 (0.09)
**Error 2**	TP	14.67 (3.49)	19.10 (0.88)	3.37 (1.50)	11.20 (2.59)	19.07 (2.05)	5.70 (2.96)
	TN	979.00(1.17)	947.43(7.08)	979.97(0.18)	978.90(0.96)	914.67(23.05)	979.73(0.52)
	F1	0.81 (0.15)	0.54 (0.06)	0.28 (0.11)	0.69 (0.12)	0.38 (0.06)	0.42 (0.18)
	MCC	0.82 (0.13)	0.59 (0.05)	0.39 (0.10)	0.70 (0.11)	0.46 (0.05)	0.50 (0.16)
**Error 3**	TP	14.70 (3.27)	19.60 (0.62)	3.90 (2.07)	6.73 (3.56)	14.47 (5.44)	3.27 (2.39)
	TN	979.17(0.70)	949.13(5.60)	979.93(0.25)	978.80(1.13)	939.77(25.56)	979.80(0.41)
	F1	0.82 (0.13)	0.56 (0.04)	0.31 (0.15)	0.46 (0.21)	0.38 (0.12)	0.26 (0.18)
	MCC	0.83 (0.11)	0.61 (0.04)	0.40 (0.17)	0.50 (0.21)	0.42(0.13)	0.34 (0.20)
**Error 4**	TP	16.10 (2.84)	19.33 (0.84)	4.30 (1.93)	7.87 (4.74)	10.00 (8.22)	3.07 (3.80)
	TN	978.70(0.88)	947.60(5.30)	979.83(0.38)	976.67(5.27)	951.43(26.44)	979.87(0.43)
	F1	0.85 (0.09)	0.54 (0.05)	0.34 (0.13)	0.48 (0.24)	0.26 (0.19)	0.22 (0.25)
	MCC	0.86 (0.08)	0.59 (0.04)	0.44 (0.13)	0.51 (0.23)	0.29(0.22)	0.28 (0.27)
**Error 5**	TP	12.47 (2.83)	17.30 (1.78)	3.17 (1.98)	4.33 (3.18)	8.27(6.59)	1.40 (1.50)
	TN	978.17(1.66)	947.10(7.17)	979.87(0.43)	977.77(1.91)	955.53(29.29)	979.70(0.60)
	F1	0.72 (0.12)	0.50 (0.07)	0.26 (0.15)	0.31 (0.18)	0.27(0.18)	0.12 (0.12)
	MCC	0.73 (0.10)	0.54 (0.07)	0.36 (0.16)	0.35 (0.19)	0.29(0.19)	0.19 (0.17)

4.4 Simulations using SKCM Gene Expression data

To generate a genotype matrix that reflects realistic dependence structures observed in real data, we extract correlation matrices from the TCGA SKCM gene expression data. Specifically, we select 1,000 and 2,000 gene expression profiles from the working dataset to estimate the correlation structures, which are then used to simulate genotype matrices via a multivariate normal distribution. Tables S6 and S7 (Appendix) exhibit performance patterns similar to those in Table 1 and Table S5. RLSS consistently achieves high TP, F1, and MCC values while maintaining low FP rates across all error types. In particular, RLSS outperforms competing methods in terms of F1 score and MCC under heavy-tailed errors; for example, under Error3 in Table S6, RLSS attains an F1 score of 0.81 (sd 0.08) and an MCC of 0.82 (sd 0.08), whereas RLP and LP perform poorly. We also note that stability selection alone does not guarantee strong performance, particularly under heavy-tailed error distributions. For example, the performance LSS, the non-robust counterpart of RLSS, deteriorates markedly as the error distribution becomes heavier-tailed. In contrast, RLSS maintains a favorable balance between true positives and false positives across all settings, with consistently high F1 scores and MCCs, highlighting its robustness and reliability under diverse error distributions and complex data structures.

Table S6: Evaluation of the homogeneous model simulated using SKCM data (n,p) = (500,1000) with 100 replicates.

		RLSS	RL	RLP	LSS	LASSO	LP
**Error 1**	TP	19.30 (0.75)	19.70 (0.02)	5.00 (2.44)	18.63 (1.27)	19.80 (1.10)	14.93 (3.28)
	TN	977.93(1.55)	797.77(75.78)	979.93(0.25)	979.53(0.73)	906.20(25.18)	979.40(0.72)
	F1	0.93 (0.04)	0.20 (0.06)	0.38 (0.16)	0.91 (0.03)	0.37 (0.07)	0.83 (0.12)
	MCC	0.93 (0.04)	0.30 (0.07)	0.47 (0.15)	0.91 (0.03)	0.46 (0.06)	0.84 (0.10)
**Error 2**	TP	15.27 (2.38)	19.97 (0.18)	3.70 (2.28)	9.33 (2.77)	19.27 (1.08)	7.03 (3.05)
	TN	978.37(1.25)	830.90(66.17)	979.43(0.90)	978.97(1.27)	910.90(20.56)	979.27(0.87)
	F1	0.82 (0.08)	0.23 (0.06)	0.29 (0.16)	0.60 (0.14)	0.37 (0.07)	0.49 (0.16)
	MCC	0.83 (0.08)	0.33 (0.06)	0.37 (0.16)	0.64 (0.12)	0.45 (0.06)	0.55 (0.13)
**Error 3**	TP	14.83 (2.61)	19.87 (0.35)	4.53 (2.03)	5.20 (2.06)	15.83 (2.57)	3.27 (2.32)
	TN	978.57(1.41)	874.53(31.08)	979.73(0.52)	978.47(1.11)	931.60(19.79)	979.53(0.78)
	F1	0.81 (0.08)	0.28 (0.04)	0.35 (0.13)	0.38 (0.13)	0.39 (0.06)	0.26 (0.17)
	MCC	0.82 (0.08)	0.38 (0.04)	0.45 (0.11)	0.44 (0.12)	0.43 (0.05)	0.34 (0.17)
**Error 4**	TP	16.10 (2.14)	19.70 (0.53)	4.40 (2.04)	7.27 (5.23)	8.07 (8.66)	3.20 (4.70)
	TN	977.90(1.30)	854.97(47.81)	979.60(0.72)	971.83(23.14)	956.90(27.33)	979.67(0.55)
	F1	0.84 (0.07)	0.26 (0.06)	0.34 (0.13)	0.41 (0.26)	0.20 (0.21)	0.22 (0.28)
	MCC	0.84 (0.07)	0.35 (0.06)	0.43 (0.12)	0.44 (0.25)	0.23 (0.23)	0.25 (0.30)
**Error 5**	TP	12.50 (2.39)	18.90 (1.30)	2.47 (1.50)	3.40 (1.67)	7.60 (3.60)	2.30 (1.80)
	TN	978.23(1.45)	838.40(76.32)	979.93(0.37)	977.57(2.43)	949.33(16.21)	977.60(2.31)
	F1	0.72 (0.09)	0.24 (0.07)	0.21 (0.12)	0.26 (0.11)	0.25 (0.09)	0.17 (0.12)
	MCC	0.73 (0.09)	0.33 (0.07)	0.32 (0.14)	0.31 (0.12)	0.25 (0.09)	0.22 (0.13)

Table S7: Evaluation of the homogeneous model simulated using SKCM data $(n, p) =$ (1000,2000) with 100 replicates.

		RLSS	RL	RLP	LSS	LASSO	LP
**Error 1**	TP	19.93 (0.25)	19.97 (0.02)	5.72 (6.64)	19.93 (0.25)	20.00 (0.00)	19.83 (0.53)
	TN	1979.33(0.66)	1769.70(79.51)	1979.92(0.16)	1978.83(0.95)	1894.30(28.50)	1979.60(0.50)
	F1	0.98 (0.02)	0.18 (0.06)	0.36 (0.34)	0.97 (0.02)	0.33 (0.07)	0.97 (0.02)
	MCC	0.98 (0.02)	0.29 (0.06)	0.44 (0.30)	0.97 (0.02)	0.44 (0.06)	0.97 (0.02)
**Error 2**	TP	19.73 (0.64)	19.10 (1.21)	4.30 (5.78)	13.17 (3.35)	19.20 (2.02)	8.30 (3.05)
	TN	1979.23(0.90)	1824.43(66.38)	1979.72(0.35)	1977.20(2.12)	1901.90(27.06)	1979.70(0.53)
	F1	0.95 (0.03)	0.23 (0.08)	0.28 (0.32)	0.72 (0.14)	0.36 (0.09)	0.56 (0.16)
	MCC	0.95 (0.03)	0.34 (0.07)	0.35 (0.30)	0.73 (0.14)	0.46 (0.08)	0.62 (0.12)
**Error 3**	TP	15.94 (4.06)	19.87 (0.10)	6.26 (6.55)	7.70 (2.45)	19.23 (1.30)	8.07 (3.15)
	TN	1979.74(0.49)	1908.13(21.14)	1979.80(0.45)	1975.60(2.39)	1907.77(26.71)	1979.57(0.77)
	F1	0.87 (0.13)	0.37 (0.05)	0.39 (0.35)	0.48 (0.13)	0.36 (0.09)	0.55 (0.15)
	MCC	0.88 (0.11)	0.46 (0.04)	0.45 (0.32)	0.49 (0.13)	0.45 (0.07)	0.61 (0.12)
**Error 4**	TP	16.62 (3.83)	19.80 (0.10)	5.05 (6.15)	7.87 (4.08)	6.23 (8.18)	1.83 (3.17)
	TN	1979.57(0.67)	1914.03(32.61)	1979.84(0.37)	1963.20(22.71)	1960.47(29.00)	1979.83(0.38)
	F1	0.88 (0.12)	0.41 (0.11)	0.32 (0.32)	0.39 (0.23)	0.16 (0.18)	0.13 (0.22)
	MCC	0.89 (0.11)	0.50 (0.09)	0.40 (0.29)	0.41 (0.23)	0.19 (0.21)	0.16 (0.25)
**Error 5**	TP	12.75 (6.00)	19.93 (0.37)	4.45 (4.75)	6.53 (2.91)	12.80 (6.50)	3.40 (3.38)
	TN	1979.58(0.70)	1878.60(54.86)	1979.87(0.43)	1976.57(2.46)	1941.67(27.05)	1979.87(0.35)
	F1	0.72 (0.24)	0.31 (0.08)	0.06 (0.07)	0.31 (0.29)	0.34 (0.15)	0.26 (0.22)
	MCC	0.76 (0.20)	0.42 (0.07)	0.12 (0.13)	0.36 (0.29)	0.39 (0.15)	0.33 (0.23)

4.5 Simulations under AR(1) correlation and heterogeneous model errors

Recall from the simulation section in the main text that a heterogeneous error model is defined as

$$y_i = 1 + \sum_{j=1}^{k} \beta_j x_{ij} + (1 + x_{ij})\epsilon_i,$$

where $k = 20$ denotes the number of important features (i.e., the total number of true positives). Model performance under AR(1) correlation and the heterogeneous error structure with $(n, p) = (1000, 2000)$ is summarized in Table S8. The performance patterns are similar to those reported in Table 1 and Table S5, with RLSS consistently emerging as a leading method across 100 replicates.

With the above data generating model, all the five errors lead to heterogeneous and heavy-tailed model errors. We can observe that RLSS consistently maintains its advantage. Under Error 1 (N(0, 1)), RLSS achieves a true positive (TP) of 19.97 (sd 0.18) and a true negative (TN) of 1979.30 (sd 0.99), which is comparable to the performance of LP (Yang et al., 2020[9]). Under Error 2 (10% $N(0, 4)$ + 90% $N(0, 1)$), RLSS attains a TP of 19.80 (sd 0.41), whereas LP achieves only 8.37 (sd 2.97). Under Error 3 (LogNormal(0,1)), RLSS records a TP of 19.47 (sd 0.63), substantially outperforming LSS, which yields 8.33 (sd 3.34). Similarly, under Error 4 (90% $N(0, 1)$ + 10% Cauchy(0,1)), RLSS achieves a TP of 19.87 (sd 0.35), compared to 8.27 (sd 8.17) for LASSO. Finally, under Error 5 ($t(2)$), RLSS attains a TP of 19.10 (sd 0.88), demonstrating stable performance, while LP drops to 1.50 (sd 2.06).

Overall, across all heterogeneous error distributions, RLSS consistently demonstrates superior and reliable performance, often outperforming or showing markedly greater stability than competing methods.

Table S8: Evaluation of the heterogeneous model under AR(1) correlation $(n, p) =$ (1000,2000) with 100 replicates.

		RLSS	RL	RLP	LSS	LASSO	LP
**Error 1**	TP	19.97 (0.18)	19.83 (0.15)	15.43 (3.32)	19.67 (0.55)	20.00 (0.00)	18.47 (1.07)
	TN	1979.30(0.99)	1888.00(1.07)	1979.90(0.31)	1978.27(1.46)	1903.77(18.10)	1979.83(0.46)
	F1	0.98 (0.03)	0.33 (0.08)	0.86 (0.12)	0.95 (0.04)	0.35 (0.06)	0.96 (0.03)
	MCC	0.98 (0.03)	0.43 (0.07)	0.87 (0.10)	0.95 (0.03)	0.45 (0.05)	0.96 (0.03)
**Error 2**	TP	19.80 (0.41)	19.70 (2.43)	11.70 (3.11)	7.00 (4.76)	19.23 (1.01)	8.37 (2.97)
	TN	1979.47(0.57)	1910.03(2.67)	1979.90(0.31)	1967.60(19.64)	1908.93(27.28)	1979.80(0.41)
	F1	0.98 (0.02)	0.37 (0.06)	0.72 (0.13)	0.38 (0.25)	0.37 (0.09)	0.57 (0.16)
	MCC	0.98 (0.02)	0.47 (0.05)	0.75 (0.11)	0.39 (0.25)	0.46 (0.07)	0.62 (0.15)
**Error 3**	TP	19.47 (0.63)	19.97 (0.18)	13.17 (2.97)	8.33 (3.34)	14.70 (5.25)	4.23 (3.38)
	TN	1979.47(0.78)	1918.10(2.23)	1979.90(0.31)	1975.70(2.35)	1930.80(27.28)	1979.93(0.25)
	F1	0.97 (0.03)	0.41 (0.09)	0.78 (0.10)	0.50 (0.16)	0.35 (0.11)	0.32 (0.22)
	MCC	0.97 (0.03)	0.50 (0.08)	0.80 (0.09)	0.51 (0.15)	0.41 (0.12)	0.41 (0.20)
**Error 4**	TP	19.87 (0.35)	19.67 (0.18)	14.00 (3.02)	9.20 (4.54)	8.27 (8.17)	2.93 (4.29)
	TN	1979.50(0.78)	1913.97(1.47)	1979.97(0.18)	1941.33(149.03)	1957.60(28.71)	1979.83(0.38)
	F1	0.98 (0.02)	0.40 (0.10)	0.81 (0.11)	0.45 (0.24)	0.23 (0.22)	0.20 (0.28)
	MCC	0.98 (0.02)	0.49 (0.08)	0.83 (0.10)	0.46 (0.24)	0.26 (0.23)	0.23 (0.30)
**Error 5**	TP	19.10 (0.88)	18.90 (2.30)	8.27 (2.74)	5.37 (1.71)	8.60 (5.46)	1.50 (2.06)
	TN	1979.47(0.68)	1911.63(3.42)	1979.97(0.18)	1971.77(7.80)	1951.73(26.35)	1979.97(0.18)
	F1	0.96 (0.03)	0.39 (0.09)	0.57 (0.13)	0.33 (0.10)	0.28 (0.16)	0.12 (0.16)
	MCC	0.96 (0.03)	0.48 (0.07)	0.63 (0.11)	0.34 (0.11)	0.30 (0.16)	0.18 (0.21)

4.6 Simulations under a higher ratio of p/n

We demonstrate the insensitivity of the proposed RLSS to the ratio of p and n. Under the homogeneous model with AR(1) correlation, building on Table 1 with $(n, p) = (1000, 2000)$ and Table S5 with $(n, p) = (500, 1000)$, we conducted additional simulations at $(n, p) = (1000, 5000)$ and $(n, p) = (500, 2500)$, reported in Tables S9 and S10, respectively. Comparing Table 1 with Table S9, RLSS maintains stable variable selection accuracy in the $p = 5n$ regime across all error distributions except Error 5 ($t(2)$), where performance slightly declines but still outperforms competing methods. For the smaller sample size ($n = 500$), all methods exhibit notable performance degradation as p increases from 1000 (Table S5) to 2500 (Table S10); however, RLSS continues to demonstrate a clear advantage over the alternatives.

Table S9: Evaluation of the homogeneous model under AR(1) correlation (n, p) = (1000,5000) with 100 replicates.

		RLSS	RL	RLP	LSS	LASSO	LP
**Error 1**	TP	20.00 (0.00)	20.00 (0.00)	13.87 (3.82)	20.00 (0.00)	20.00 (0.00)	19.93 (0.25)
	TN	4977.43(1.65)	4617.80(3.44)	4980.00(0.00)	4979.70(0.53)	4883.53(41.81)	4979.93(0.25)
	F1	0.94 (0.04)	0.12 (0.05)	0.80 (0.14)	0.99 (0.01)	0.32 (0.08)	1.00 (0.01)
	MCC	0.94 (0.03)	0.24 (0.06)	0.82 (0.12)	0.99 (0.01)	0.43 (0.07)	1.00 (0.01)
**Error 2**	TP	19.57 (0.86)	19.47 (0.38)	11.27 (3.35)	16.83 (2.18)	19.97 (0.18)	13.97 (3.51)
	TN	4979.43(0.73)	4800.87(17.15)	4979.93(0.25)	4979.63(0.49)	4888.90(31.46)	4979.97(0.18)
	F1	0.97 (0.03)	0.26 (0.12)	0.71 (0.13)	0.90 (0.07)	0.32 (0.07)	0.81 (0.14)
	MCC	0.98 (0.03)	0.38 (0.11)	0.74 (0.11)	0.91 (0.06)	0.43 (0.06)	0.83 (0.11)
**Error 3**	TP	19.27 (0.94)	19.97 (0.18)	12.67 (3.74)	10.27 (3.45)	18.30 (2.90)	6.90 (3.75)
	TN	4979.80(0.41)	4896.50(3.02)	4979.93(0.25)	4979.27(0.87)	4905.77(31.09)	4979.77(0.57)
	F1	0.98 (0.03)	0.34 (0.05)	0.76 (0.15)	0.65 (0.16)	0.34 (0.08)	0.48 (0.21)
	MCC	0.98 (0.03)	0.44 (0.04)	0.78 (0.12)	0.68 (0.15)	0.44 (0.06)	0.55 (0.17)
**Error 4**	TP	19.40 (0.77)	19.60 (2.33)	12.97 (3.72)	5.13 (3.61)	6.90 (8.27)	3.27 (5.13)
	TN	4979.73(0.52)	4831.97(7.71)	4979.90(0.31)	4976.40(8.11)	4952.90(39.40)	4979.80(0.48)
	F1	0.98 (0.02)	0.24 (0.09)	0.77 (0.14)	0.34 (0.22)	0.16 (0.17)	0.22 (0.30)
	MCC	0.98 (0.02)	0.36 (0.08)	0.79 (0.12)	0.39 (0.22)	0.20 (0.20)	0.25 (0.32)
**Error 5**	TP	16.23 (2.19)	16.97 (0.18)	7.30 (3.04)	5.60 (2.74)	12.70 (7.18)	2.30 (2.58)
	TN	4979.70(0.65)	4888.23(4.97)	4979.97(0.18)	4978.27(1.36)	4929.50(33.46)	4979.83(0.46)
	F1	0.88 (0.07)	0.33 (0.08)	0.51 (0.18)	0.40 (0.16)	0.27 (0.13)	0.18 (0.19)
	MCC	0.89 (0.07)	0.44 (0.07)	0.58 (0.16)	0.45 (0.17)	0.33 (0.15)	0.25 (0.22)

Table S10: Evaluation of the homogeneous model under AR(1) correlation $(n, p) = (500,2500)$ with 100 replicates.

		RLSS	RL	RLP	LSS	LASSO	LP
**Error 1**	TP	19.13 (1.11)	19.97 (0.18)	3.23 (1.99)	16.43 (1.96)	20.00 (0.00)	12.67 (3.21)
	TN	2477.07(2.05)	2388.13(11.46)	2479.97(0.18)	2479.73(0.45)	2380.60(34.85)	2479.80(0.41)
	F1	0.91 (0.05)	0.31 (0.03)	0.27 (0.14)	0.89 (0.07)	0.30 (0.08)	0.76 (0.12)
	MCC	0.91 (0.05)	0.42 (0.02)	0.38 (0.13)	0.90 (0.06)	0.41 (0.06)	0.78 (0.10)
**Error 2**	TP	9.40 (3.77)	18.70 (1.44)	2.23 (1.63)	5.87 (2.54)	17.87 (2.05)	4.43 (1.83)
	TN	2479.50(0.78)	2389.53(8.18)	2480.00(0.00)	2479.67(0.55)	2414.03(26.25)	2479.83(0.46)
	F1	0.61 (0.19)	0.29 (0.03)	0.19 (0.13)	0.43 (0.15)	0.36 (0.08)	0.35 (0.12)
	MCC	0.65 (0.17)	0.39 (0.03)	0.30 (0.15)	0.51 (0.13)	0.44 (0.06)	0.45 (0.10)
**Error 3**	TP	7.37 (3.48)	19.33 (0.76)	2.57 (1.74)	2.77 (1.91)	12.37 (5.86)	1.80 (1.54)
	TN	2479.67(0.61)	2392.70(15.76)	2479.77(0.50)	2479.23(0.90)	2427.80(37.47)	2479.87(0.35)
	F1	0.51 (0.19)	0.31 (0.06)	0.21 (0.14)	0.22 (0.14)	0.28 (0.14)	0.15 (0.13)
	MCC	0.57 (0.17)	0.42 (0.05)	0.31 (0.16)	0.29 (0.17)	0.33 (0.15)	0.24 (0.17)
**Error 4**	TP	10.10 (4.80)	18.83 (2.26)	2.40 (2.01)	3.00 (3.40)	6.70 (7.79)	1.30 (2.20)
	TN	2479.63(0.67)	2401.43(24.42)	2479.90(0.31)	2478.47(1.87)	2455.30(37.08)	2479.83(0.59)
	F1	0.63 (0.22)	0.33 (0.07)	0.20 (0.15)	0.22 (0.21)	0.18 (0.17)	0.10 (0.17)
	MCC	0.67 (0.18)	0.43 (0.04)	0.29 (0.17)	0.28 (0.22)	0.20 (0.20)	0.15 (0.21)
**Error 5**	TP	10.50 (2.10)	16.43 (2.36)	1.27 (1.36)	1.63 (1.35)	6.60 (5.93)	0.67 (0.96)
	TN	2476.93(1.55)	2407.37(28.91)	2479.93(0.25)	2479.00(1.14)	2463.03(19.47)	2479.93(0.37)
	F1	0.62 (0.10)	0.32 (0.09)	0.11 (0.12)	0.14 (0.11)	0.24 (0.18)	0.06 (0.08)
	MCC	0.63 (0.09)	0.40 (0.07)	0.18 (0.17)	0.20 (0.15)	0.27 (0.19)	0.12 (0.14)

4.7 Sensitivity analysis results

In this section, the following tables present results illustrating the sensitivity of model performance to the subsampling ratio q and the threshold θ. Specifically, we considered: (1) two subsampling ratios, $q = 70\%$ and 80%; and (2) three threshold values, $\theta = 50\%, 80\%, 90\%$. Table S11, S12, S13, S14 show results under the homogeneous data structure while Table S15 presents results under heterogeneous structure. In addition, Table S16 and S17 provide results when non-zero regression coefficients are generated from Unif(0.2,0.8).

Overall, the results are not sensitive to reasonable choices of q and θ. For example, a comparison of Tables S11 and S13 shows that, under $(n, p) = (500, 1000)$, the best F1 score and MCC are similar for $q = 0.7$ and 0.8, indicating that subsamples with such high sampling ratios are sufficiently representative of the original dataset. Within each table in Tables S11—-S15, performance is poor when the selection ratio is 50%, as many false signals are selected. In contrast, increasing the selection ratio to 80% or 90% substantially improves variable selection performance, with some evidence suggesting that a 90% threshold performs slightly better than 80%. The sensitivity analyses further support the choice of the 90% selection stability threshold adopted in Yang et al. (2020).[9].

Table S11: Sensitivity analysis under the homogeneous model under AR(1) correlation with subsampling ratio $q = 70\%$ and selection threshold $\theta \in \{50\%,80\%,90\%\}$ for (n,p)= (500,1000). The highest F1 and MCC values are shown in boldface.

		RLSS			LSS		
		50%	80%	90%	50%	80%	90%
**Error 1**	TP	20.00 (0.00)	19.97 (0.18)	19.77 (0.50)	20.00 (0.00)	19.80 (0.55)	19.07 (1.08)
	TN	902.40 (17.21)	968.50 (3.31)	976.37 (1.94)	870.50 (6.82)	975.10 (1.83)	979.43 (0.73)
	F1	0.35 (0.05)	0.78 (0.05)	0.91 (0.04)	0.27 (0.01)	0.89 (0.04)	**0.96 (0.04)**
	MCC	0.28 (0.03)	0.79 (0.04)	0.91 (0.04)	0.37 (0.01)	0.89 (0.04)	**0.96 (0.04)**
**Error 2**	TP	19.33 (1.03)	17.20 (1.71)	14.73 (2.70)	18.03 (1.52)	11.37 (2.92)	6.73 (2.98)
	TN	955.43 (8.73)	977.23 (1.99)	979.23 (1.04)	794.13(50.13)	970.27 (3.86)	978.63 (1.33)
	F1	0.61 (0.08)	**0.86 (0.06)**	0.82 (0.09)	0.17 (0.04)	0.55 (0.14)	0.46 (0.16)
	MCC	0.65 (0.06)	**0.86 (0.06)**	0.83 (0.08)	0.25 (0.05)	0.55 (0.14)	0.51 (0.15)
**Error 3**	TP	19.17 (0.95)	16.30 (2.55)	13.27 (3.29)	17.57 (1.94)	10.23 (2.69)	5.90 (2.23)
	TN	955.33 (11.32)	977.43 (2.10)	979.40 (1.00)	798.77(22.98)	967.93(4.89)	978.03(1.35)
	F1	0.61 (0.09)	**0.84 (0.08)**	0.77 (0.12)	0.16 (0.03)	0.49 (0.12)	0.41 (0.12)
	MCC	0.65 (0.07)	**0.84 (0.07)**	0.79 (0.10)	0.24 (0.04)	0.48 (0.13)	0.46 (0.11)
**Error 4**	TP	19.63 (0.61)	18.03 (1.73)	16.07 (2.77)	19.13 (1.59)	12.37 (4.75)	8.03 (4.65)
	TN	946.43 (17.03)	975.90 (3.12)	978.73 (1.44)	537.43(347.46)	944.83(71.65)	975.40(11.34)
	F1	0.56 (0.11)	**0.86 (0.06)**	**0.86 (0.08)**	0.13 (0.08)	0.48 (0.24)	0.49 (0.23)
	MCC	0.61 (0.09)	**0.86 (0.06)**	**0.86 (0.07)**	0.18 (0.13)	0.49 (0.23)	0.52 (0.22)
**Error 5**	TP	18.27 (1.53)	14.83 (2.09)	12.17 (2.18)	17.30 (1.90)	8.60 (2.70)	4.83 (2.53)
	TN	938.23 (11.54)	973.40 (3.33)	978.17 (1.21)	723.30(161.51)	961.50(33.25)	977.47(2.33)
	F1	0.46 (0.06)	**0.72 (0.07)**	0.71 (0.08)	0.13 (0.04)	0.41 (0.14)	0.34 (0.16)
	MCC	0.52 (0.05)	0.71 (0.07)	**0.72 (0.07)**	0.20 (0.07)	0.40 (0.13)	0.38 (0.16)

Table S12: Sensitivity analysis under the homogeneous model under AR(1) correlation with subsampling ratio $q = 70\%$ and selection threshold $\theta \in \{50\%,80\%,90\%\}$ for $(n, p)=$ (1000,2000). The highest F1 and MCC values are shown in boldface.

		RLSS			LSS		
		50%	80%	90%	50%	80%	90%
**Error 1**	TP	20.00 (0.00)	20.00 (0.00)	20.00 (0.00)	20.00 (0.00)	20.00 (0.00)	20.00 (0.00)
	TN	1912.93(15.24)	1970.47(4.17)	1976.70(2.09)	1788.80(8.10)	1971.47(2.99)	1979.13(0.97)
	F1	0.38 (0.05)	0.81 (0.07)	0.93 (0.04)	0.17 (0.01)	0.83 (0.05)	**0.98 (0.02)**
	MCC	0.43 (0.04)	0.84 (0.06)	0.93 (0.04)	0.29 (0.01)	0.84 (0.04)	**0.98 (0.02)**
**Error 2**	TP	20.00 (0.00)	19.97 (0.18)	19.93 (0.25)	19.97 (0.18)	19.83 (0.38)	18.77 (1.19)
	TN	1944.40(14.66)	1976.23(2.62)	1979.10(0.80)	1733.00(13.25)	1966.70(3.40)	1978.17(1.32)
	F1	0.55 (0.10)	0.92 (0.05)	**0.98 (0.02)**	0.14 (0.01)	0.75 (0.05)	0.92 (0.04)
	MCC	0.55 (0.08)	0.93 (0.06)	**0.98 (0.02)**	0.26 (0.01)	0.77 (0.04)	0.92 (0.04)
**Error 3**	TP	20.00 (0.00)	20.00 (0.00)	19.97 (0.18)	19.87 (0.43)	17.40 (2.22)	13.43 (3.46)
	TN	1963.50(6.04)	1978.10(1.40)	1979.47(0.73)	1652.63(85.66)	1961.90(8.53)	1976.83(1.68)
	F1	0.72 (0.07)	0.96 (0.03)	**0.99 (0.02)**	0.11 (0.02)	0.64 (0.10)	0.73 (0.14)
	MCC	0.66 (0.06)	0.96 (0.03)	**0.99 (0.02)**	0.22 (0.03)	0.66 (0.10)	0.73 (0.14)
**Error 4**	TP	20.00 (0.00)	20.00 (0.00)	19.97 (0.18)	19.60 (0.72)	14.63 (4.21)	10.37 (5.12)
	TN	1960.13(5.97)	1977.50(1.74)	1979.17(1.09)	999.53(684.50)	1869.90(196.55)	1966.80(30.44)
	F1	0.67 (0.07)	0.94 (0.04)	**0.98 (0.02)**	0.06 (0.04)	0.41 (0.25)	0.53 (0.25)
	MCC	0.71(0.06)	0.94 (0.04)	**0.98 (0.02)**	0.12 (0.09)	0.44 (0.24)	0.55 (0.24)
**Error 5**	TP	19.97 (0.18)	19.43 (0.90)	18.87 (1.14)	18.80 (1.47)	12.80 (3.73)	8.70 (3.77)
	TN	1959.37(5.03)	1978.03(1.47)	1979.17(0.87)	1470.33(330.65)	1941.50(65.27)	1974.90(4.35)
	F1	0.66 (0.06)	0.94 (0.04)	**0.95 (0.04)**	0.08 (0.03)	0.42 (0.15)	0.50 (0.18)
	MCC	0.63 (0.04)	0.94 (0.04)	**0.95 (0.04)**	0.17 (0.05)	0.44 (0.15)	0.52 (0.17)

Table S13: Sensitivity analysis under the homogeneous model under AR(1) correlation with subsampling ratio $q = 80\%$ and selection threshold $\theta \in \{50\%,80\%,90\%\}$ for (n, p)= (500,1000). The highest F1 and MCC values are shown in boldface.

		RLSS			LSS		
		50%	80%	90%	50%	80%	90%
**Error 1**	TP	20.00 (0.00)	20.00 (0.00)	19.97 (0.18)	20.00 (0.00)	19.80 (0.41)	18.97 (0.89)
	TN	883.47 (18.01)	956.33 (6.08)	969.53 (2.53)	873.37 (9.24)	975.43 (2.14)	979.33 (0.80)
	F1	0.30 (0.04)	0.63 (0.06)	0.79 (0.04)	0.27 (0.02)	0.89 (0.05)	**0.96 (0.03)**
	MCC	0.25 (0.02)	0.67 (0.05)	0.81 (0.03)	0.38 (0.02)	0.90 (0.04)	**0.96 (0.03)**
**Error 2**	TP	19.70 (0.60)	18.87 (1.38)	17.67 (1.92)	19.63 (0.49)	15.23 (1.98)	10.43 (2.57)
	TN	925.60 (16.03)	970.43 (3.66)	975.87 (2.75)	841.87(12.15)	972.73 (2.86)	978.93 (1.23)
	F1	0.43 (0.07)	0.78 (0.07)	**0.85 (0.08)**	0.22 (0.01)	0.72 (0.06)	0.66 (0.12)
	MCC	0.51 (0.06)	0.79 (0.07)	**0.85 (0.08)**	0.32 (0.02)	0.71 (0.07)	0.68 (0.11)
**Error 3**	TP	19.63 (0.56)	18.40 (1.52)	17.13 (2.27)	17.80 (1.71)	10.67 (3.55)	6.83 (3.17)
	TN	935.80 (23.35)	971.63 (5.93)	976.80 (2.43)	803.13(30.16)	969.40 (4.24)	978.27 (1.87)
	F1	0.50 (0.11)	0.80 (0.08)	**0.85 (0.06)**	0.17 (0.03)	0.51 (0.14)	0.46 (0.18)
	MCC	0.56 (0.09)	0.80 (0.07)	**0.85 (0.06)**	0.25 (0.05)	0.51 (0.14)	0.51 (0.16)
**Error 4**	TP	19.83 (0.46)	18.90 (1.18)	18.07 (1.46)	18.70 (1.44)	11.83 (5.48)	7.80 (5.71)
	TN	929.90 (22.94)	970.77 (6.55)	977.03 (2.51)	581.17(292.73)	956.37(37.45)	977.57(3.72)
	F1	0.46 (0.10)	0.80 (0.09)	**0.88 (0.06)**	0.12 (0.07)	0.48 (0.22)	0.47 (0.27)
	MCC	0.54 (0.07)	0.81 (0.08)	**0.88 (0.06)**	0.18 (0.11)	0.49 (0.22)	0.51 (0.26)
**Error 5**	TP	19.13 (1.01)	17.23 (1.65)	15.13 (2.26)	17.30 (1.29)	8.47 (2.54)	4.03 (2.03)
	TN	929.00 (12.67)	970.33 (4.19)	975.83 (2.29)	720.80(119.76)	963.93(11.89)	977.27 (1.80)
	F1	0.43 (0.05)	0.74 (0.07)	**0.77 (0.07)**	0.13 (0.04)	0.40 (0.14)	0.29 (0.13)
	MCC	0.50 (0.05)	0.74 (0.07)	**0.77 (0.07)**	0.20 (0.06)	0.39 (0.14)	0.33 (0.13)

Table S14: Sensitivity analysis under the homogeneous model under AR(1) correlation with subsampling ratio $q = 80\%$ and selection threshold $\theta \in \{50\%, 80\%, 90\%\}$ for (n,p)= (1000,2000). The highest F1 and MCC values are shown in boldface.

		RLSS			LSS		
		50%	80%	90%	50%	80%	90%
**Error 1**	TP	20.00 (0.00)	20.00 (0.00)	20.00 (0.00)	20.00 (0.00)	20.00 (0.00)	20.00 (0.00)
	TN	1893.60(14.13)	1958.50(6.03)	1971.53(3.28)	1786.70(11.56)	1971.37(3.60)	1978.83(1.29)
	F1	0.32 (0.03)	0.66 (0.06)	0.83 (0.05)	0.17 (0.01)	0.83 (0.06)	**0.97 (0.03)**
	MCC	0.38 (0.03)	0.62 (0.05)	0.84 (0.05)	0.29 (0.01)	0.84 (0.05)	**0.97 (0.03)**
**Error 2**	TP	20.00 (0.00)	20.00 (0.00)	20.00 (0.00)	19.77 (0.50)	14.33 (4.87)	10.77 (5.79)
	TN	1930.33(12.74)	1969.50(4.71)	1975.80(2.25)	1078.03(67.94)	1748.07(48.71)	1915.50(27.92)
	F1	0.46 (0.07)	0.80 (0.07)	**0.91 (0.05)**	0.07 (0.04)	0.38 (0.26)	0.51 (0.30)
	MCC	0.48 (0.05)	0.83 (0.05)	**0.92 (0.04)**	0.13 (0.09)	0.41 (0.26)	0.53 (0.28)
**Error 3**	TP	20.00 (0.00)	20.00 (0.00)	20.00 (0.00)	19.47 (0.82)	13.70 (3.73)	8.87 (3.89)
	TN	1953.70(9.65)	1974.53(3.23)	1977.97(1.54)	1467.60(18.22)	1953.90(11.96)	1976.27(1.98)
	F1	0.62 (0.08)	0.80 (0.07)	**0.95 (0.03)**	0.08 (0.02)	0.47 (0.15)	0.53 (0.19)
	MCC	0.59 (0.06)	0.80 (0.06)	**0.95 (0.03)**	0.17 (0.05)	0.49 (0.15)	0.54 (0.18)
**Error 4**	TP	20.00 (0.00)	20.00 (0.00)	19.97 (0.19)	19.53 (0.90)	14.53 (4.83)	10.03 (5.67)
	TN	1950.67 (9.40)	1974.47(3.30)	1978.07(1.63)	970.07(647.06)	1912.53(85.72)	1974.87(5.58)
	F1	0.59 (0.08)	0.88 (0.06)	**0.94 (0.06)**	0.06 (0.04)	0.42 (0.25)	0.56 (0.27)
	MCC	0.57 (0.06)	0.89 (0.05)	**0.95 (0.05)**	0.11 (0.08)	0.45 (0.24)	0.57 (0.26)
**Error 5**	TP	19.97 (0.18)	19.80 (0.48)	19.64 (0.69)	19.27 (1.05)	14.10 (3.42)	9.47 (4.17)
	TN	1947.23 (7.53)	1973.17(3.37)	1977.21(1.86)	1453.60(385.24)	1948.50(40.09)	1974.40(8.30)
	F1	0.56 (0.06)	0.85 (0.06)	**0.92 (0.05)**	0.08 (0.03)	0.48 (0.14)	0.54 (0.21)
	MCC	0.55 (0.05)	0.88 (0.06)	**0.93 (0.05)**	0.17 (0.05)	0.50 (0.14)	0.55 (0.21)

Table S15: Sensitivity analysis under the heterogeneous model under AR(1) correlation with subsampling ratio $q = 70\%$ and selection threshold $\theta \in \{50\%, 80\%, 90\%\}$ for (n, p)= (1000,2000). The highest F1 and MCC values are shown in boldface.

		RLSS			LSS		
		50%	80%	90%	50%	80%	90%
**Error 1**	TP	19.97 (0.18)	19.93 (0.25)	19.83 (0.38)	18.87 (0.82)	12.53 (2.66)	7.23 (2.53)
	TN	1954.33(10.57)	1976.63(2.11)	1978.93(0.83)	1527.37(145.27)	1954.17(10.34)	1975.27(2.41)
	F1	0.62 (0.09)	0.92 (0.04)	**0.97 (0.02)**	0.08 (0.02)	0.44 (0.13)	0.45 (0.14)
	MCC	0.60 (0.07)	0.93 (0.03)	**0.97 (0.02)**	0.17 (0.03)	0.46 (0.13)	0.46 (0.13)
**Error 2**	TP	20.00 (0.00)	19.97 (0.18)	19.87 (0.35)	18.17 (1.68)	9.80 (3.47)	5.37 (2.88)
	TN	1961.30(4.52)	1977.57(1.55)	1979.17(0.83)	1320.97(263.55)	1940.37(18.85)	1974.23(2.84)
	F1	0.69 (0.05)	0.94 (0.03)	**0.98 (0.02)**	0.06 (0.02)	0.31 (0.13)	0.34 (0.17)
	MCC	0.64 (0.04)	0.94 (0.03)	**0.98 (0.02)**	0.13 (0.05)	0.32 (0.14)	0.35 (0.18)
**Error 3**	TP	19.83 (0.38)	19.80 (0.41)	19.70 (0.47)	19.00 (1.41)	13.23 (2.60)	8.17 (2.79)
	TN	1966.73(4.95)	1978.90(1.21)	1979.70(0.60)	1514.13(138.22)	1954.60(9.28)	1975.40(2.44)
	F1	0.75 (0.07)	0.97 (0.03)	**0.99 (0.02)**	0.08 (0.02)	0.46 (0.12)	0.49 (0.15)
	MCC	0.79 (0.06)	0.97 (0.03)	**0.99 (0.02)**	0.17 (0.04)	0.48 (0.12)	0.51 (0.14)
**Error 4**	TP	20.00 (0.00)	20.00 (0.00)	19.83 (0.38)	19.60 (1.00)	14.27 (4.00)	9.00 (4.25)
	TN	1962.13(6.00)	1977.50(1.93)	1979.40(0.81)	909.73(702.59)	1783.00(340.65)	1939.00(125.02)
	F1	0.70 (0.07)	0.94 (0.04)	**0.98 (0.02)**	0.06 (0.04)	0.34 (0.24)	0.43 (0.24)
	MCC	0.75 (0.06)	0.94 (0.04)	**0.98 (0.02)**	0.11 (0.09)	0.38 (0.23)	0.45 (0.23)
**Error 5**	TP	19.97 (0.18)	19.67 (0.55)	19.23 (1.10)	17.70 (1.29)	9.67 (2.94)	5.37 (2.51)
	FP	1961.97(6.99)	1978.03(1.67)	1979.50(0.68)	1347.53(170.98)	1939.33(13.55)	1973.10(3.37)
	F1	0.70 (0.08)	0.95 (0.04)	**0.97 (0.04)**	0.06 (0.01)	0.29 (0.12)	0.32 (0.13)
	MCC	0.75 (0.07)	0.95 (0.04)	**0.97 (0.03)**	0.12 (0.03)	0.31 (0.12)	0.33 (0.13)

To further confirm the utility of using 90% as the selection-frequency cutoff, we conducted an additional sensitivity analysis under varying signal strengths by generating 20 nonzero coefficients from Unif$(0.2, 0.8)$, as shown in Table S16 and Table S17. The results suggest that a 90% threshold provides a robust and stable cutoff across different signal settings. Our analysis is consistent with the superior performance of using 90% cutoff for selection frequency reported in Yang et al. (2020)[9].

More importantly, we emphasize that RLSS should be viewed primarily as a method for prioritizing or ranking genomic features according to their stability measures. This framework provides flexibility in selecting the final set of top-ranked genomic features when domain expertise or prior biological knowledge suggests that a cutoff other than 90% may be more appropriate. It is also well suited to applications in which practitioners are more interested in examining the robust ranking for genomic features of interest rather than identifying a fixed set of selected features.

Table S16: Sensitivity analysis under the homogeneous model under AR(1) correlation with subsampling ratio $q = 70\%$ and selection threshold $\theta \in \{50\%,80\%,90\%\}$ for (n, p)= (500,1000). The non-zero coefficients are generated from unif(0.2,0.8). The highest F1 and MCC values are shown in boldface.

		RLSS			LSS		
		50%	80%	90%	50%	80%	90%
**Error 1**	TP	20.00 (0.00)	19.83 (0.38)	19.73 (0.52)	20.00 (0.00)	19.63 (0.61)	19.20 (0.71)
	TN	896.77(18.71)	966.67(5.28)	976.33(2.22)	753.57(54.26)	972.13(3.16)	978.87(1.20)
	F1	0.33 (0.05)	0.75 (0.08)	0.91 (0.05)	0.16 (0.03)	0.83 (0.06)	**0.95 (0.03)**
	MCC	0.43 (0.05)	0.77 (0.07)	0.91 (0.05)	0.25 (0.04)	0.84 (0.06)	**0.95 (0.03)**
**Error 2**	TP	19.60 (0.67)	18.53 (1.38)	18.07 (1.53)	19.57 (0.73)	17.63 (1.33)	16.20 (1.71)
	TN	943.83(11.45)	975.77(2.14)	979.03(1.19)	784.70(27.89)	971.23(3.15)	978.77(1.19)
	F1	0.53 (0.08)	0.87 (0.06)	**0.92 (0.06)**	0.17 (0.02)	0.76 (0.06)	0.86 (0.06)
	MCC	0.58 (0.06)	0.87 (0.06)	**0.92 (0.06)**	0.27 (0.02)	0.77 (0.06)	0.87 (0.06)
**Error 3**	TP	19.47 (0.73)	18.43 (1.17)	17.37 (1.71)	19.43 (0.77)	16.00 (2.05)	13.47 (2.99)
	TN	962.47(7.32)	978.80(1.00)	979.70(0.47)	738.10(62.37)	967.90(5.56)	978.40(1.79)
	F1	0.69 (0.08)	**0.93 (0.03)**	**0.92 (0.05)**	0.14 (0.03)	0.67 (0.09)	0.76 (0.13)
	MCC	0.72 (0.07)	**0.93 (0.03)**	**0.92 (0.05)**	0.24 (0.03)	0.67 (0.09)	0.77 (0.12)
**Error 4**	TP	19.57 (0.57)	18.63 (1.16)	17.77 (1.25)	19.73 (0.52)	15.80 (3.19)	12.53 (4.47)
	TN	955.30 (16.62)	977.17(3.37)	979.17(1.32)	564.50(297.37)	947.80(44.78)	976.70(4.15)
	F1	0.64 (0.12)	0.90 (0.06)	**0.92 (0.04)**	0.12 (0.05)	0.57 (0.22)	0.69 (0.20)
	MCC	0.67 (0.10)	0.90 (0.06)	**0.92 (0.04)**	0.19 (0.10)	0.58 (0.21)	0.70 (0.20)
**Error 5**	TP	18.90 (1.16)	17.50 (1.31)	16.63 (1.33)	18.43 (1.30)	14.03 (2.59)	11.03 (2.97)
	TN	945.70 (11.92)	974.70(2.78)	978.10(1.42)	684.10(106.09)	962.33(16.32)	977.77(1.70)
	F1	0.53 (0.07)	0.82 (0.06)	**0.86 (0.05)**	0.12 (0.03)	0.57 (0.13)	0.65 (0.14)
	MCC	0.58 (0.06)	0.82 (0.06)	**0.86 (0.05)**	0.19 (0.05)	0.57 (0.13)	0.67 (0.13)

Table S17: Sensitivity analysis under the homogeneous model under AR(1) correlation with subsampling ratio q = 80% and selection threshold $\theta \in \{50\%,80\%,90\%\}$ for (n, p)= (500,1000). The non-zero coefficients are generated from unif(0.2,0.8). The highest F1 and MCC values are shown in boldface.

		RLSS			LSS		
		50%	80%	90%	50%	80%	90%
**Error 1**	TP	19.97 (0.18)	19.93 (0.25)	19.77 (0.43)	20.00 (0.00)	19.73 (0.45)	19.10 (0.96)
	TN	881.07(20.32)	957.53(7.56)	970.77(4.35)	776.33(32.48)	972.73(3.25)	979.03(0.96)
	F1	0.29 (0.04)	0.65 (0.08)	0.81 (0.07)	0.17 (0.02)	0.84 (0.06)	**0.95 (0.03)**
	MCC	0.39 (0.04)	0.68 (0.07)	0.82 (0.06)	0.27 (0.03)	0.85 (0.05)	**0.95 (0.03)**
**Error 2**	TP	19.77 (0.43)	19.43 (0.68)	19.17 (0.83)	19.77 (0.43)	18.07 (1.31)	16.63 (1.96)
	TN	926.00(15.91)	968.47(4.46)	974.93(2.07)	768.17(27.53)	969.97(2.44)	978.30(1.49)
	F1	0.43 (0.08)	0.77 (0.07)	**0.88 (0.04)**	0.16 (0.02)	0.75 (0.05)	0.87 (0.07)
	MCC	0.51 (0.06)	0.78 (0.06)	**0.89 (0.04)**	0.26 (0.02)	0.76 (0.05)	0.87 (0.07)
**Error 3**	TP	19.47 (0.78)	18.60 (1.38)	18.20 (1.49)	18.93 (1.28)	15.80 (2.02)	13.97 (2.17)
	TN	947.37(11.31)	973.57(2.96)	977.57(1.63)	756.07(37.43)	968.17(3.34)	978.67(1.18)
	F1	0.55 (0.08)	0.83 (0.05)	**0.90 (0.05)**	0.15 (0.02)	0.66 (0.07)	0.79 (0.08)
	MCC	0.60 (0.07)	0.83 (0.05)	**0.89 (0.05)**	0.24 (0.03)	0.67 (0.08)	0.79 (0.08)
**Error 4**	TP	19.57 (0.68)	18.83 (1.05)	18.57 (1.19)	19.30 (0.88)	14.47 (4.22)	11.50 (4.97)
	TN	948.10(16.50)	973.63(4.06)	977.63(2.03)	508.40(283.04)	907.93(155.94)	972.03(20.99)
	F1	0.57 (0.10)	0.84 (0.06)	**0.91 (0.05)**	0.10 (0.05)	0.48 (0.24)	0.61 (0.24)
	MCC	0.62 (0.08)	0.84 (0.06)	**0.91 (0.05)**	0.15 (0.10)	0.49 (0.24)	0.63 (0.22)
**Error 5**	TP	19.20 (0.85)	18.27 (1.23)	17.53 (1.38)	18.70 (1.37)	14.37 (2.50)	11.67 (3.06)
	TN	942.20(11.34)	971.90(4.64)	976.50(2.10)	691.93(108.29)	964.83(9.43)	977.60(1.99)
	F1	0.51 (0.07)	0.79 (0.07)	**0.86 (0.05)**	0.12 (0.03)	0.59 (0.11)	0.68 (0.13)
	MCC	0.56 (0.06)	0.80 (0.07)	**0.85 (0.05)**	0.20 (0.05)	0.59 (0.11)	0.69 (0.13)

5 Real Data Analysis

5.1 The Overlapping Table

Table S18: Identification results for SKCM and eQTL data. The numbers of gene expressions identified by different methods and their overlaps.

**SKCM**	RLSS	RL	RLP	LSS	LASSO	LP
RLSS	17	6	5	6	9	4
RL		28	15	0	5	0
RLP			21	1	5	1
LSS				18	12	9
LASSO					32	15
LP						15
**eQTL**	RLSS	RL	RLP	LSS	LASSO	LP
RLSS	14	14	7	11	14	11
RL		85	9	12	43	15
RLP			12	6	9	8
LSS				12	12	10
LASSO					44	15
LP						15

5.1.1 SKCM

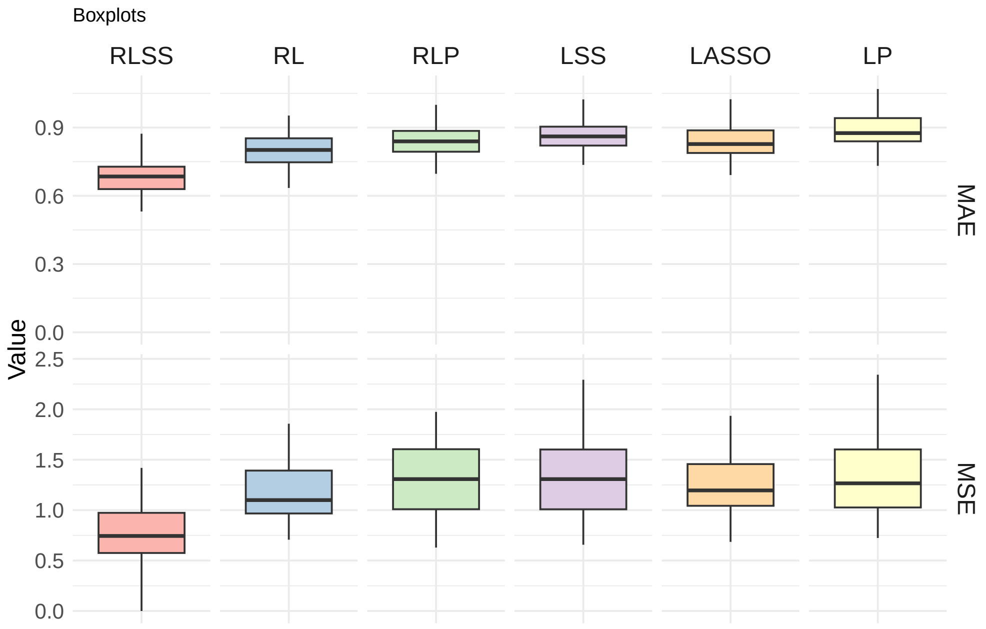

Figure S3: Prediction performance of all methods for SKCM. The boxplots display the distributions of prediction errors across 100 replicates for each method, with predicted mean absolute error (MAE; top panel) and predicted mean squared error (MSE; bottom panel).

Table S19: Identification results for all methods under comparison for SKCM data.

Gene*	RLSS	RL	RLP	LSS	LASSO	LP
ALDH4A1		0.013				
ANTXR2		-0.018				
ATAD2B		0.165				
BOLA1				-0.117		
BTNL9		-0.148	-0.174			
C13orf21	0.171			0.142	0.100	0.163
C14orf61	-0.143	-0.101	-0.127		-0.025	
C1ORF216					0.126	
CCDC142	-0.036		-0.071			
CDKN1A				0.062	0.044	0.053
COQ9	-0.318			-0.117		
DKFZP586C1324	0.103			0.131	0.117	0.142
DNASE1L1		0.142				
DNLC2B				-0.171	-0.186	-0.170
DOCK11			0.014			
DSTYK		0.129				
EBF2					-0.145	-0.270
FAM222A-AS1					0.100	
FAM49A		0.010	-0.023			
FLJ13621		-0.051				
FLJ20373					-0.084	
FMNL2	-0.161	-0.168			-0.043	
H2A					-0.177	
HBD					-0.077	-0.131
HPE2				0.073		
IER5	0.027			0.082	0.111	
INPP5K	-0.003	0.159	0.113			
JMS		-0.126	-0.147			
KBF2			-0.219	-0.126	-0.053	-0.134
KCNG4					-0.065	
KCTD16					-0.095	-0.018
LBR			-0.040			
LINC00847					-0.132	
LOC100240735					0.035	
LOC100506644		-0.127	-0.045		-0.032	
LOC105371602					-0.031	-0.084
LOC115722	0.032	0.098	0.077			
LOC116242		0.029	0.119			
LOC148709				0.149	0.101	
LOC90374	0.087					

Continued on the next page

Table S19: Continued from the previous page.

Gene*	RLSS	RL	RLP	LSS	LASSO	LP
LOC91492				0.049		
LOC92224		0.027				
MYO1C			0.000			
NEIL2		0.048				
PARD6G	-0.039	0.018	0.044			
PER1				-0.182		
PITPNA	0.146			0.135	0.151	0.122
QRSL1					-0.026	
RABIF		-0.064				
RBFA	0.029					
RYBP				-0.161	-0.027	-0.095
SDPR	-0.148	-0.106	-0.112		-0.045	
SEL1L3	-0.094	-0.182				
SEMA3A	-0.369			-0.307	-0.135	-0.147
SHF				0.085		
SLC8A1		0.041	0.007		-0.037	
SMPX					-0.070	-0.092
SP3		0.136	0.157			
STAG2		0.058	0.009			
TDRG1				-0.271	-0.210	
TFR2	0.099				0.097	
TMEM159				0.160	0.134	0.177
TTC28-AS1					-0.062	-0.359
ZEB1		-0.125	-0.091			
ZFHX3		-0.084				
ZNF41		0.119	0.145			
ZNF586		-0.067	-0.034			

5.1.2 The eQTL data

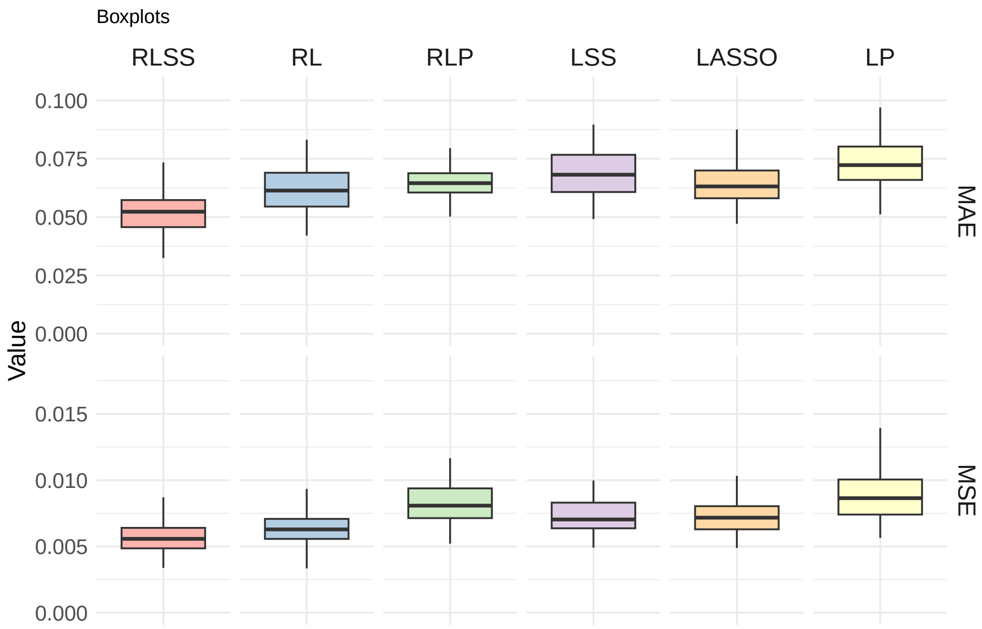

Figure S4: Prediction performance of all methods for the eQTL data. The boxplots display the distributions of prediction errors across 100 replicates for each method, with predicted mean absolute error (MAE; top panel) and predicted mean squared error (MSE; bottom panel).

Table S20: Identification results for all methods under comparison for eQTL data.

Gene*	RLSS	RL	RLP	LSS	LASSO	LP
1367491_at		-0.023				
1367530_at		0.036				
1367547_at		-0.036				
1367671_at	-0.103	-0.106			-0.104	
1367890_at		-0.001				
1368136_at		0.031				
1369995_at		-0.073				
1370188_at		-0.049				
1370360_at		-0.051				
1371351_at		-0.012				
1371463_at		0.009				
1371495_at		-0.014				
1371581_at		0.027				
1371610_at					0.063	
1371614_at	0.115	0.099		0.093	0.061	0.095
1371661_at	0.168	0.068		0.140	0.074	0.087
1371740_at		0.027			0.045	
1371956_at		-0.020				
1372083_at		-0.039				
1372220_at		-0.082				
1372248_at	0.087	0.047	0.127	0.103	0.043	0.068
1372394_at		-0.055			-0.071	
1372446_at		-0.089			-0.044	
1372453_at		0.159			0.146	
1372582_at		0.048				
1372674_at		0.074			0.032	
1372713_at		0.003				
1373011_at		0.017				
1373015_at		0.036				
1373016_at		0.083		0.074	0.118	
1373095_at	0.072	0.021	0.113	0.085	-0.004	0.077
1373294_at		-0.010				
1373373_at		-0.006				
1373376_at		0.026				
1373409_at		0.050			0.069	
1373474_at		-0.094			-0.104	
1373770_at		0.032				
1373809_at		-0.004				
1373846_at		0.024				
1373848_at		0.011			0.014	

Continued on the next page

Table S20: Continued from the previous page.

Gene*	RLSS	RL	RLP	LSS	LASSO	LP
1373887_at	0.078	0.061			0.054	0.029
1373896_at	0.167	0.114	0.149	0.203	0.112	0.182
1373912_at		0.018				
1373968_at		-0.107			-0.108	
1374056_at		0.056				
1374075_at		-0.086			-0.085	
1374094_at		-0.034				
1374151_at		-0.013				
1375019_at		0.006			0.025	
1375896_at		-0.014			0.005	0.027
1377194_a_at		0.035			0.038	
1377701_at		0.092			0.066	
1382065_at		0.044			0.051	
1382139_at		0.011				
1382223_at		0.072	0.090		0.057	0.040
1383081_at		0.095			0.075	0.008
1383520_at		-0.041			-0.060	
1384022_at	-0.179	-0.122		-0.200	-0.144	
1388491_at	0.156	0.046	0.125	0.136	0.097	0.118
1388615_at		0.005				
1388679_at		0.016				
1388690_at		-0.011				
1389057_at	0.092	0.137		0.090	0.108	0.048
1389130_at	0.199	0.104	0.194	0.219	0.110	0.188
1389357_at		0.096				
1389476_at		-0.046			-0.073	
1389910_at	0.275	0.208	0.284	0.261	0.230	0.219
1389968_at		0.013				
1390142_at		0.024			0.016	
1391412_at			0.055			
1391484_at		-0.004	0.054		0.013	0.030
1392468_at		-0.064				
1392469_at		0.027			0.018	
1392918_at		0.060			0.060	
1392930_at		0.088			0.058	
1393000_at			-0.007			
1398340_at		0.032				
1398389_at		0.036				
1398615_at		0.021			0.048	
1398916_at	0.119	0.086		0.118	0.099	0.091
1398934_at		-0.061				

Continued on the next page

Table S20: Continued from the previous page.

Gene*	RLSS	RL	RLP	LSS	LASSO	LP
1399067_at		-0.090			-0.123	
1378935_at		-0.018				
1382835_at	0.025	0.027	0.009		0.035	
1383749_at		-0.001				
1385043_at		0.034			0.037	
1390401_at			0.014			
1393543_at		-0.020				
1394399_at		0.005			0.016	

References

[1] Hansheng Wang, Guodong Li, and Guohua Jiang. Robust regression shrinkage and consistent variable selection through the lad-lasso. *Journal of Business & Economic Statistics*, 25(3):347–355, 2007.

[2] Tong Tong Wu and Kenneth Lange. Coordinate descent algorithms for lasso penalized regression. *The Annals of Applied Statistics*, 2(1):224–244, 2008.

[3] Jie Ren, Yinhao Du, Shaoyu Li, Shuangge Ma, Yu Jiang, and Cen Wu. Robust network-based regularization and variable selection for high-dimensional genomic data in cancer prognosis. *Genetic epidemiology*, 43(3):276–291, 2019.

[4] Cen Wu and Shuangge Ma. A selective review of robust variable selection with applications in bioinformatics. *Briefings in bioinformatics*, 16(5):873–883, 2015.

[5] Jie Ren, L. Jung, Yinhao Du, Cen Wu, Yu Jiang, and Junhao Liu. *regnet: Network-Based Regularization for Generalized Linear Models*, 2022. URL `https://CRAN.R-project.org/package=regnet`. R package version 1.0.

[6] Jerome H Friedman, Trevor Hastie, and Rob Tibshirani. Regularization paths for generalized linear models via coordinate descent. *Journal of statistical software*, 33:1–22, 2010.

[7] Nicolai Meinshausen and Peter Bühlmann. Stability selection. *Journal of the Royal Statistical Society: Series B (Statistical Methodology)*, 72(4):417–473, 2010.

[8] Robert Tibshirani. Regression shrinkage and selection via the lasso. *Journal of the Royal Statistical Society: Series B (Methodological)*, 58(1):267–288, 1996.

[9] Songshan Yang, Jiawei Wen, Scott T Eckert, Yaqun Wang, Dajiang J Liu, Rongling Wu, Runze Li, and Xiang Zhan. Prioritizing genetic variants in gwas with lasso using permutation-assisted tuning. *Bioinformatics*, 36(12):3811–3817, 2020.

[10] Kun Fan, Srijana Subedi, Vishmi Dissanayake, and Cen Wu. Robust Bayesian high-dimensional variable selection and inference with the horseshoe family of priors. *Computational Statistics & Data Analysis*, page 108358, 2026.

[11] Lan Wang, Yichao Wu, and Runze Li. Quantile regression for analyzing heterogeneity in ultra-high dimension. *Journal of the American Statistical Association*, 107(497): 214–222, 2012.
